# Preoperative CT-derived Hounsfield unit values and mechanical complications after thoracolumbar or lumbar instrumented fusion: a systematic review and meta-analysis

**DOI:** 10.3389/fsurg.2026.1909831

**Published:** 2026-10-07

**Authors:** Zhibo Song, Bo Zhou, Ni Bi, Li Zhang, Zhi Zhao, Yingsong Wang

**Affiliations:** Department of Orthopedic Surgery, The Second Affiliated Hospital of Kunming Medical University, Kunming, Yunnan, China

**Keywords:** cage subsidence, computed tomography, hounsfield unit, mechanical complications, meta-analysis, proximal junctional kyphosis, screw loosening, spinal fusion

## Abstract

**Background/objectives:**

Mechanical complications after thoracolumbar or lumbar instrumented fusion remain a major concern, especially in patients with poor bone quality. CT-derived Hounsfield unit (HU) values may offer an accessible marker of local bone strength, but the evidence is inconsistent. We evaluated its association with postoperative mechanical complications.

**Methods:**

PubMed, Embase, Web of Science, Scopus, the Cochrane Library, and Google Scholar were searched for studies of adults undergoing instrumented fusion reporting HU and postoperative mechanical outcomes. The primary outcome was overall mechanical complications. Random-effects models were used; continuous per-HU odds ratios, dichotomous low-versus-high estimates, and hazard ratios were analyzed separately.

**Results:**

Thirty-five retrospective studies were included. In twelve reporting an adjusted per-HU odds ratio, each one-unit increase carried lower odds of mechanical complications (OR 0.983; 95% CI, 0.978–0.989; *P* < 0.001), with moderate heterogeneity (I^2^ = 46.6%) and a 95% prediction interval of 0.974–0.993; per 10 HU the pooled OR was 0.846 (95% CI, 0.802–0.891). The estimate was stable on leave-one-out analysis (range 0.982–0.984) and after excluding the one study at high risk of bias (OR 0.984; 95% CI, 0.980–0.988). Outcome-specific estimates were directionally consistent but imprecise (screw loosening, k = 3, OR 0.980, 95% CI 0.949–1.012; cage subsidence, k = 5, OR 0.982, 95% CI 0.971–0.993). Of 25 studies comparing lower- with higher-HU groups, all placed lower HU at higher risk, but thresholds ranged from approximately 71–369 HU; the 11 with an odds ratio reported by the study or reconstructable from its data were summarized descriptively rather than pooled.

**Conclusions:**

Lower preoperative HU is associated with a higher risk of postoperative mechanical complications. The per-unit association was small but significant; low-versus-high comparisons were directionally consistent but rested on disparate thresholds and were not combined. HU is best used as an opportunistic marker that prompts further bone-health evaluation and individualized planning, rather than as a validated risk-stratification tool, a fixed per-unit estimate, or a universal threshold. Standardized HU measurement, consistent outcome definitions, and prospective validation are needed.

**Systematic Review Registration:**

https://www.crd.york.ac.uk/PROSPERO/view/CRD420261376259, PROSPERO, CRD420261376259.

## 1 Introduction

Instrumented thoracolumbar and lumbar fusion is among the most common spinal procedures, yet mechanical failure remains its defining limitation. Across pooled cohorts, cage subsidence affects roughly a third of patients and screw loosening about a quarter. Together with proximal junctional kyphosis and failure, pseudarthrosis, and implant failure, these events are leading drivers of revision surgery, disability, and cost (1). Of the factors that determine whether instrumentation holds, host bone quality is among the most influential, and it is one of the few that can be known before surgery.

Reliable measurement is the harder problem. Dual-energy x-ray absorptiometry (DXA), the conventional standard, is systematically confounded in fusion candidates, because osteophytes, endplate sclerosis, aortic calcification, and scoliosis all inflate lumbar areal density. In postmenopausal women, spinal DXA identifies osteoporosis in only 17.1% of patients versus 46.4% by quantitative CT, with degenerative change present in nearly every discordant case (2). The conventional test is thus least trustworthy in precisely the patients who most need an accurate answer.

The Hounsfield unit (HU) measured on a routine preoperative CT has emerged as an opportunistic alternative. It adds no scan, cost, or radiation, and it tracks closely with quantitative CT. The supporting evidence accrued quickly. HU outperforms the DXA T-score for predicting screw loosening (area under the curve 0.67 vs. 0.57) (3); lower HU independently predicts complications, with the odds rising about 1.7-fold per 25-HU decrease (4); and a meta-analysis has proposed a 135-HU osteoporosis cut-off (5). On the surface, the question looks settled.

Beneath that apparent consensus, the field has fixed on a single aim: pinning down a predictive HU “threshold.” The result is a set of irreconcilable cut-points. Reported values include 135 HU for osteoporosis (5), 120 HU or below for proximal junctional failure (6), and 116, 126, 198, and 151 HU for loosening, subsidence, and junctional failure within a single synthesis (1). These figures disagree more than threefold and shift with every spinal level, region of interest, and outcome definition, which leaves no usable bedside target. Two deeper problems follow. The first is conceptual: treating HU as a diagnostic threshold misplaces its real role, since it is a freely available signal that flags who needs closer scrutiny, not a cut-off meant to replace DXA. The second is evidentiary: the chain that runs from observing low HU, to acting on it, to actually reducing mechanical failure has never been demonstrated. It has only been assumed. The field has accumulated associations without settling how HU should be used.

What the field needs, therefore, is not another threshold but a synthesis that confronts this interpretive gap. Such a synthesis should ask why a universal cut-point cannot be derived from current evidence, reposition HU from a diagnostic threshold to an opportunistic screening signal and risk-stratifier within multifactorial planning, and make the untested step between association and action explicit. We undertook this systematic review and meta-analysis with that purpose. Rather than add another number to the literature, we set out to clarify how a routinely available signal can make preoperative decision-making more deliberate and more individualized, while keeping the limits of that signal in view.

## 2 Materials and methods

### 2.1 Study design and registration

This meta-analysis was designed to evaluate the association between preoperative CT-derived Hounsfield unit values and postoperative mechanical complications after thoracolumbar or lumbar instrumented fusion. Reporting followed the PRISMA 2020 statement, with the observational meta-analysis reported in line with the MOOSE (Meta-analysis Of Observational Studies in Epidemiology) guideline and the low-versus-high (descriptive) synthesis following the Synthesis Without Meta-analysis (SWiM) reporting items. The review was registered with PROSPERO (CRD420261376259) on 23 April 2026. Registration coincided with the search and screening stage rather than preceding it, and the review is therefore reported as retrospectively registered. The protocol (version 1.0, dated 20 April 2026) fixed the review question, eligibility criteria, outcomes, and analysis framework, and these were not revised once results became available. The population, exposure, comparator, and outcome framework was used to guide study selection and data synthesis (7).

### 2.2 Eligibility criteria

Studies were eligible if they met the following criteria. The population included adult patients undergoing thoracolumbar or lumbar instrumented spinal fusion. The exposure was preoperative CT-derived HU value measured at the vertebral body, pedicle, screw trajectory, upper instrumented vertebra, adjacent vertebra, endplate, or surgically relevant spinal level (8). The comparator included higher HU values, non-low-HU groups, or alternative HU strata or cut-off groups within the same study. The outcomes were postoperative mechanical-related adverse events, including overall mechanical complications, pedicle screw loosening, cage subsidence, proximal junctional kyphosis or proximal junctional failure, implant or instrumentation failure, pseudarthrosis or nonunion, and revision or reoperation related to mechanical failure. These elements define the review's PICOS framework: Population, adults undergoing thoracolumbar or lumbar instrumented fusion; Intervention/Exposure, preoperative CT-derived Hounsfield unit value; Comparator, higher-HU or non-low-HU reference groups; Outcomes, postoperative mechanical complications (overall and by type); and Study design, observational cohort and case-control studies. This framework was applied throughout study selection and synthesis, and the review is reported in accordance with PRISMA 2020, MOOSE, and SWiM.

Retrospective cohort studies, retrospective case–control studies, and retrospective observational studies were considered eligible when sufficient quantitative data were available. Studies were excluded if they did not involve spinal instrumentation or fusion, did not report CT-derived HU values, did not evaluate postoperative mechanical outcomes, or lacked extractable effect estimates or sufficient data for quantitative synthesis. Reviews, editorials, conference abstracts without usable data, case reports, animal studies, biomechanical-only studies, and duplicate reports were excluded.

### 2.3 Literature search

A systematic search covered PubMed, Embase, Web of Science, Scopus, and the Cochrane Library, matching the databases named in the registered protocol. Google Scholar was screened separately as a gray-literature source (first 200 results), and reference lists of relevant reviews and included studies were checked by backward and forward citation searching (9). The search strategy combined terms related to CT-derived Hounsfield units, spinal fusion, internal fixation, and postoperative mechanical complications. No restriction was placed on surgical approach at the search stage in order to capture OLIF, PLIF, TLIF, posterior fusion, and other instrumented fusion procedures (10).

Both free-text keywords and Medical Subject Headings were used for these concepts. All databases were last searched on 23 April 2026. The full PubMed search strategy is provided in the Supplementary Material (Supplementary Methods S1); the complete protocol and the strategies for all databases are available in the PROSPERO registration record (CRD420261376259).

### 2.4 Study selection

All retrieved records were imported into a reference-management file, and duplicates were removed before screening. Titles and abstracts were screened independently by two reviewers (Z.S. and B.Z.), who then assessed full texts against the predefined inclusion and exclusion criteria. Reports that were clearly unrelated to CT HU values, spinal instrumentation, or postoperative mechanical outcomes were excluded at the title-and-abstract stage. Inter-rater agreement at the full-text stage was almost perfect (Cohen's *κ* = 0.90), and any disagreements were resolved by discussion and, where needed, adjudication by a senior author.

### 2.5 Data extraction

Data were extracted independently by two reviewers (Z.S. and B.Z.) using a predesigned, piloted data extraction sheet, and any discrepancies were reconciled by discussion against the source article. The extracted information included first author, publication year, country or region, study design, sample size, patient population, surgical approach, spinal level, pathology, follow-up duration, HU measurement site, HU measurement method, outcome definition, effect measure, adjusted model, point estimate, 95% confidence interval, and *P* value.

For HU measurement, the anatomical site was recorded as reported in the original study. These sites included vertebral body, L1, L3, L4, L1–L4, L1–L5, pedicle, screw trajectory, endplate, adjacent vertebra, segmental vertebra, upper instrumented vertebra, and other surgically relevant regions. When several HU values were reported, the value most closely linked to the surgical construct or the analyzed mechanical outcome was extracted. If the study reported both univariable and multivariable results, the adjusted estimate was preferred.

All extracted effect estimates, sample sizes, and event counts were checked against the full text of the source article by both reviewers before analysis. Estimates were taken from the tables of the source article; where the text and a table of the same article disagreed, the tabulated estimate, which could be checked against its standard error, *P* value, and confidence interval, was used (40). Where a study reported several models for the same predictor, the estimate from the model adjusted for age was used (31), and hazard ratios reported for continuous and for dichotomised HU by the same study were tabulated separately (37). Two reports of one overlapping cohort [(29, 50) were treated as a single study represented by the later and larger report. For the low-versus-high comparison, an odds ratio was displayed only when the study reported an odds ratio for the dichotomised HU variable or when a crude 2 × 2 table could be reconstructed exactly from reported counts or from sensitivity and specificity that resolve to integer event counts (Supplementary Table S12); studies without such data, without a confidence interval, or with a three-level HU classification contributed directional evidence only (Supplementary Table S5). Two studies (20, 28) reported regression coefficients whose direction was inconsistent with their own group-level findings, most plausibly because the modelled event was the favourable outcome; their estimates are shown as reported (Section 3.3) and were not pooled.

### 2.6 Outcomes

The primary outcome was overall postoperative mechanical-related complications after thoracolumbar or lumbar instrumented fusion. This outcome included any reported mechanical failure or mechanical adverse event associated with the implant, fusion construct, cage, screw fixation, junctional segment, or revision caused by mechanical failure.

Secondary outcomes were specific mechanical complications, including pedicle screw loosening, cage subsidence or endplate-related complications, and proximal junctional kyphosis or proximal junctional failure. These outcomes were analyzed separately when sufficient extractable data were available. PJK/PJF analyses were treated as exploratory when the number of studies was small. The composite primary outcome, prespecified in the registered protocol, is a summary across mechanistically distinct events and was interpreted as such; outcome-specific estimates are reported alongside it and were given priority in the clinical interpretation.

### 2.7 Risk of bias assessment

Risk of bias was assessed independently by two reviewers (Z.S. and B.Z.) using the Quality in Prognosis Studies (QUIPS) tool, with disagreements resolved by discussion. The evaluated domains included study participation, attrition, prognostic factor measurement, outcome measurement, confounding, statistical analysis and reporting, and overall judgment. Each domain was rated as low, moderate, or high risk. Particular attention was paid to confounding because all included studies were retrospective and adjustment strategies varied across studies.

Bodies of evidence started at Low because all included studies were observational, and could be downgraded further for serious risk of bias, inconsistency, indirectness, or suspected publication bias. For inconsistency, a high I^2^ was not treated as an automatic downgrade; instead, the decision considered whether the heterogeneity reflected divergent effect directions or, alternatively, methodological differences in HU measurement site and outcome definition around a consistent direction of effect.

### 2.8 Statistical analysis

Effect estimates were synthesized using random-effects models because clinical and methodological heterogeneity was expected across studies. Adjusted estimates were preferred when available. To avoid inappropriate pooling across different effect measures and HU scales, continuous OR/AOR estimates were analyzed as the main adjusted-effect model, dichotomous low-HU versus high-HU estimates were analyzed as a secondary binary model, and HR-based estimates were not combined with OR/AOR estimates. Only two studies reported Cox-model hazard ratios for time-to-event outcomes. Because their outcomes, HU measurement sites, and adjustment strategies differed and the number of studies fell below the prespecified threshold for pooling, these estimates were handled by narrative description, as specified in the PROSPERO protocol. To retain their evidence contribution, both studies were also carried in the low-versus-high synthesis as directional evidence. Results were synthesized on the logarithmic scale and then back-transformed to pooled effect estimates with 95% confidence intervals.

Where sufficient data allowed, exploratory analyses stratified by complication type (screw loosening; cage subsidence or endplate-related events; proximal junctional kyphosis or failure, listed at study level without pooling) were performed and are reported in the Supplementary Material.

Pooled effects are reported with 95% prediction intervals and *τ*^2^ to convey the spread of plausible true effects (11), and heterogeneity was examined through subgroup, sensitivity, and risk-of-bias-stratified analyses (Sections 3.5–3.8).

### 2.9 Heterogeneity exploration and sensitivity analyses

To explore the heterogeneity observed in the main analysis, prespecified subgroup analyses were performed by HU measurement site, surgical approach, spinal region, and outcome category. Omnibus between-subgroup differences were evaluated for measurement site, surgical approach, and spinal region with the test of moderators (QM) from a mixed-effects meta-regression with a common between-study variance; outcome-specific estimates were summarized separately because only two to five studies contributed to each category. Because two of the four HU-site subgroups contained a single study, the vertebral-body and pedicle/trajectory subgroups were additionally compared directly. Exploratory meta-regression on publication year and mean follow-up duration was used to probe heterogeneity; with twelve studies these models were underpowered and are summarized qualitatively.

Sensitivity analyses included (i) comparison across estimators (REML with Hartung–Knapp–Sidik–Jonkman adjustment, REML with Wald-type intervals, DerSimonian–Laird, and fixed-effect models; Supplementary Table S2); (ii) leave-one-out and leave-one-outcome-out re-pooling (Supplementary Table S7); and (iii) restriction to studies not rated at high overall risk of bias on the QUIPS tool (Supplementary Table S3). 95% prediction intervals were reported alongside 95% confidence intervals, and I^2^ was calculated as (Q−df)/Q. Small-study effects were examined with a contour-enhanced funnel plot, Egger's regression test, and Duval–Tweedie trim-and-fill (L0 estimator, with the side of imputation selected from the data). Subgroup analyses were prespecified but interpreted as hypothesis-generating; no formal multiple-comparison correction was applied. In an additional robustness check, the continuous model was re-pooled after restricting to studies that adjusted for age, and separately for age and sex (Supplementary Table S10). All statistical analyses were performed in R (version 4.3.3) using the metafor package (version 4.4-0); forest and funnel plots were generated in Python (matplotlib, version 3.10.9). The study-level data and analysis code are available from the corresponding author.

### 2.10 Differences from the registered protocol

A small number of protocol elements were not carried out as planned, in each case because of the nature of the available evidence rather than the direction of the findings. Every eligible study proved to be retrospective, which left the planned comparison of prospective and retrospective designs without data. Primary studies also reported HU on incompatible scales, so the intended harmonisation to per-10-HU and per-standard-deviation increments could not be completed; continuous effects are instead reported per 1-HU, with a per-10-HU conversion supplied for clinical reading. The Hartung-Knapp-Sidik-Jonkman interval applies to both the per-1-HU estimate and its per-10-HU rescaling, the latter being a linear transformation on the log-odds scale. Gray-literature searching was confined to Google Scholar rather than the wider set of sources named in the protocol. The risk-of-bias instrument was changed from the Newcastle–Ottawa Scale named in the protocol to the QUIPS tool, which is purpose-built for prognostic-factor reviews and better captures the prognostic-factor-measurement and confounding domains central to this question; the change concerned the appraisal instrument only. The primary random-effects model was changed from the registered DerSimonian–Laird estimator to REML with the Hartung–Knapp–Sidik–Jonkman adjustment, which yields better-calibrated confidence intervals for the small, heterogeneous set of studies analyzed; DerSimonian–Laird and Paule–Mandel estimates are reported as sensitivity analyses (Supplementary Table S2) and were materially unchanged. Finally, the diagnostic-accuracy synthesis prespecified in the protocol (pooled sensitivity and specificity with bivariate and hierarchical SROC models) could not be performed, because the included studies defined low-HU thresholds at incompatible vertebral sites across a wide range (approximately 71–369 HU) and did not report comparable threshold-level 2 × 2 accuracy data; threshold evidence is therefore summarized descriptively rather than pooled. None of these changes touched the review question, the eligibility criteria, or the primary outcome.

## 3 Results

### 3.1 Study selection

The database search identified 2,347 records. After removal of 10 duplicates, 2,337 records were screened by title and abstract, of which 2,234 were excluded; the remaining 103 full-text reports were assessed for eligibility and 68 were excluded for the reasons summarized in Figure 1, leaving 35 retrospective studies that met all eligibility criteria and were included in the qualitative and quantitative synthesis. The reasons for exclusion at the full-text stage are summarized by category in the PRISMA flow diagram (Figure 1).

**Figure 1:**
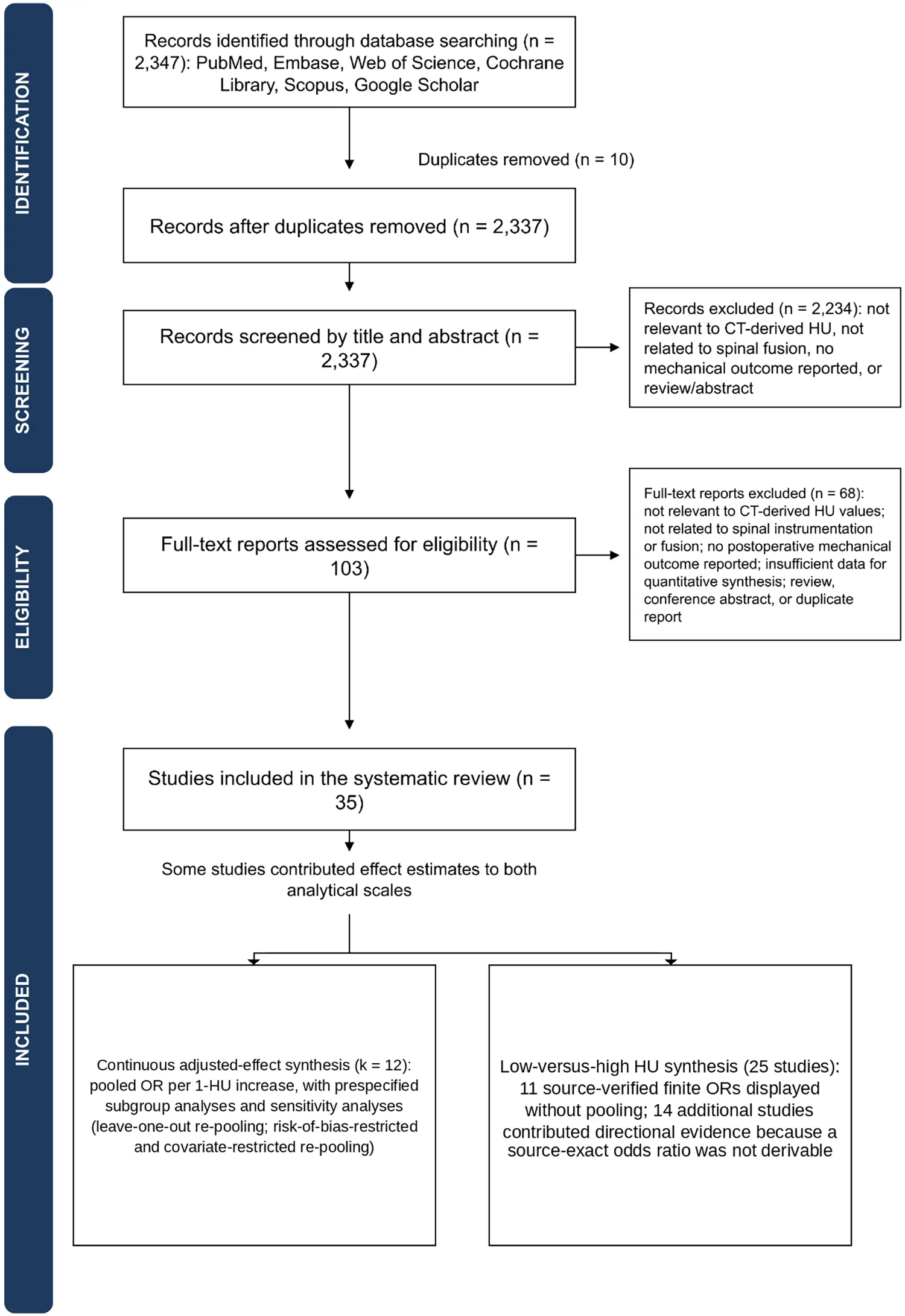
PRISMA 2020 flow diagram of study selection. A total of 2,347 records were identified through database searching. After removal of 10 duplicate records, 2,337 records were screened by title and abstract, of which 2,234 were excluded. A total of 103 full-text reports were assessed for eligibility, and 68 reports were excluded for the following reasons: not relevant to CT-derived Hounsfield unit values, not related to spinal instrumentation or fusion, no postoperative mechanical outcome reported, insufficient data for quantitative synthesis, or review/conference abstract/duplicate report. Finally, 35 studies were included in the qualitative and quantitative synthesis.

The 35 studies in Table 1 entered the synthesis through different routes, and most contributed to more than one analysis. The main continuous adjusted-effect model drew on 12 estimates from 12 studies. Chen et al. [(30); cage subsidence] and Chen et al. [(31); pedicle screw loosening] are separate reports from different author groups and patient cohorts, and each contributed one estimate. The secondary low-versus-high comparison was not combined into a summary estimate; the 11 studies with an odds ratio that could be reported or reconstructed exactly from the source are displayed without a pooled diamond, and 14 further studies contributed directional evidence only. Nine studies did not enter the continuous per-HU pool and contributed instead to the low-versus-high or directional synthesis: Levy et al. (43), Arimura et al. (27), Lee et al. (17), Liu et al. (35), Muheremu et al. (18), Pennington et al. (37), Gong et al. (16), Ding et al. (32), and Zhang et al. (28). Among these, Liu et al. (35) and Pennington et al. (37) contributed Cox-model hazard ratios, Arimura et al. (27) an HU-to-pentosidine-ratio estimate, and the others threshold-based or group-level evidence. Shu et al. (29) contributed group-level directional evidence only, and Wang et al. (47) contributed an adjusted per-HU estimate to the continuous model; the remaining binary estimates overlap with studies already in the continuous model. Liu et al. (35) and Pennington et al. (37) reported Cox-model hazard ratios, which were handled narratively (Section 2.8); Pennington's threshold-based hazard ratio was retained as directional evidence, and neither hazard ratio was combined with odds ratios. One further study, Yang et al. (48), met the eligibility criteria and is listed in Table 1, but its adjusted estimate could not be placed on either the continuous or the dichotomous scale; it therefore contributes to the descriptive synthesis only and enters no pooled model. A study-by-analysis attribution matrix is provided in Supplementary Table S5.

**Table 1:** Characteristics of studies included in the quantitative meta-analysis.

Study	Country/region	Design	Follow-up (months)	Patients (n)	Events (n)	Surgery/approach	Pathology	Main outcome	HU measurement site
Zhou 2021 (15)	China	Retrospective cohort	28.2	76	16	OLIF (SA)	Degenerative	Cage subsidence	Vertebral body
Gong 2022 (16)	China	Retrospective cohort	20.8	73	12 (surgical segment-level: 93 segments/12 events)	OLIF	Lumbar degenerative disease	Cage subsidence	L1
Lee 2022 (17)	South Korea	Retrospective case-control	NR	92	46	Posterior thoracic fusion	Mixed	Screw loosening	UIV vertebral body
Muheremu 2022 (18)	China	Retrospective cohort	NR	162	68	Lumbosacral fusion	Degenerative	Screw loosening (sacral)	Sacrum (S1)
Li 2022 (19)	China	Retrospective cohort	12	56	NR (vertebra-level: 112 vertebrae/40 loosened)	OLIF	Degenerative	Screw loosening	Holding plane
Xie 2022 (20)	China	Retrospective cohort	18	279	82	TLIF	Degenerative	Cage subsidence	Vertebral body
Zhang 2022 (21)	China	Retrospective case–control	24.2	166	83	Posterior	Deformity	PJK	UIV only
Hiyama 2022 (22)	Japan	Retrospective cohort	12	59	20	LLIF + PPS	Degenerative lumbar	Cage subsidence	Endplate (cranial/caudal)
Wu 2023 (23)	China	Retrospective case series	12	61	25 endplate violations (segment-level: 107 segments); 45 delayed subsidence	OLIF	Degenerative lumbar	Intraoperative endplate violation	Endplate (epiphyseal ring + central)
Li 2023 (24)	China	Retrospective cohort	12.2	56	NR (vertebra-level: 112 vertebrae/40 loosened)	OLIF	Degenerative	Screw loosening	Screw insertion
Yao 2023 (25)	Taiwan, China	Retrospective cohort	25.3	198	70	MI-TLIF	Degenerative	CS/SL	L1
Zhao 2023 (26)	China	Retrospective cohort	12	90	33	PLIF/TLIF	Degenerative	Screw loosening	Trajectory
Arimura 2024 (27)	Japan	Retrospective case-control	12	35	14	Mixed	Degenerative	Screw loosening	L4
Zhang 2024 (28)	China	Retrospective cohort	NR	103	31 (screw-level: 412 screws/55 loosened)	PLF/PLIF	Lumbar degenerative disease	Screw loosening	Pedicle (ROI2)
Shu 2024 (29)	China	Retrospective cohort	28.4	242	53	Lumbar fusion (PSF)	Degenerative	Screw loosening	L1–L5 average
Chen 2025 (30)	China	Retrospective cohort	12	112	29	OLIF	Degenerative	Cage subsidence	L1
Chen 2025 (31)	China	Retrospective cohort	12.6	73	17	PLIF/TLIF	Degenerative	Screw loosening	Pedicle
Ding 2025 (32)	China	Retrospective cohort	12	226	102	ULIF/TLIF	Lumbar degenerative disease	Cage subsidence	L1-L4, L4, L5
Geng 2025 (33)	China	Retrospective case-control	24	164	26	Posterior	Deformity	PJK/PJF	L1–5
Koo 2025 (34)	South Korea	Retrospective cohort	12	70	20	OLIF	Degenerative	Cage subsidence	Adjacent vertebra
Liu 2025 (35)	China	Retrospective cohort	11.4	314	42	OLIF	Degenerative	Cage subsidence	Segmental
Moridaira 2025 (36)	Japan	Retrospective cohort	75.7	46	17	Posterior	ASD	PJF	UIV
Pennington 2025 (37)	USA	Retrospective cohort	24	76	15	Posterior	Deformity	PJK	UIV
Sun 2025 (38)	China	Retrospective cohort	12	720	230 (training cohort, *n* = 620; plus 100 external validation)	PLIF	Degenerative	Cage subsidence	L3 vertebral
Yamamoto 2025 (39)	Japan	Retrospective observational	12	47	9 (screw-level: 212 screws/25 loosened)	TLIF/PLIF	Degenerative	Screw loosening	Trajectory
Yamauchi 2025 (40)	Japan	Retrospective cohort	24	129	33	Posterior	Deformity	Mechanical complications	Lumbar
Yuan 2025 (41)	China	Retrospective cohort	24	153	72	Posterior	DLS	Distal loosening	L1
Wang 2025 (42)	China	Retrospective cohort	8.6	84	29	PLIF	Degenerative lumbar	Cage subsidence	L4 vertebra
Levy 2026 (43)	USA	Retrospective cohort	12.2	114	72	TLIF	Degenerative lumbar	Cage subsidence	Endplate + Vertebral
Ke 2026 (44)	China	Retrospective cohort	12	285	61	PLIF	Degenerative	Screw loosening	S1/L1
Zhao 2026 (45)	China	Two-center retrospective study	NR	316	71	MIDLIF + CBT	Degenerative lumbar	Cage subsidence	Vertebral (L1-L5 average)
Du 2026 (46)	China	Retrospective study	12	275	56	PLIF	Degenerative lumbar	Pedicle screw loosening	Pedicle + L1 vertebra
Wang 2026 (47)	China	Retrospective cohort	8.8	129	45	PLIF	Lumbar degenerative disease	Cage subsidence	L1-L4
Yang 2026 (48)	China	Retrospective cohort	12	313	45	Posterior	Degenerative	Screw loosening	Screw trajectory + vertebral
Zhan 2026 (49)	China	Retrospective cohort	18	160	60	PLIF/TLIF	Degenerative	Cage subsidence	L1-L4

HU, Hounsfield unit; MIDLIF, midline lumbar interbody fusion; MI-TLIF, minimally invasive transforaminal lumbar interbody fusion; NR, not reported; OLIF, oblique lumbar interbody fusion; PJK, proximal junctional kyphosis; PJF, proximal junctional failure; PLF, posterolateral fusion; PLIF, posterior lumbar interbody fusion; SA, stand-alone; TLIF, transforaminal lumbar interbody fusion; UIV, upper instrumented vertebra; ULIF, unilateral biportal endoscopic lumbar interbody fusion. Sample sizes and event counts were verified against each source article; where a study analyzed screws, vertebrae, or surgical segments rather than patients, the analysis unit and unit-level counts are given in parentheses. Shu et al. (29) and Shu 2023 report overlapping cohorts from one center and are represented by the later and larger report; Zhang et al. (28) analyzed 412 screws in 103 patients.

### 3.2 Characteristics of included studies

The 35 included studies were all retrospective in design, including retrospective cohort studies, retrospective case–control studies, retrospective observational studies, and one two-center retrospective study. The studies were conducted mainly in China, Japan, South Korea, and the United States. The surgical procedures covered a broad range of thoracolumbar or lumbar fusion techniques, including OLIF, PLIF, TLIF, MI-TLIF, posterior fusion, and mixed or specialized approaches (12). The target populations mainly involved patients with degenerative lumbar disease, adult spinal deformity, or related spinal conditions requiring instrumented fusion.

Follow-up time varied across studies, ranging from approximately 8.6 months to 75.7 months where reported. Several studies reported a minimum or mean follow-up of around 12 months, while a few studies had longer observation periods. The main postoperative outcomes included cage subsidence, pedicle screw loosening, proximal junctional kyphosis or failure, distal loosening, intraoperative endplate violation, and broader mechanical complications. CT-derived HU values were measured at different anatomical sites, including the vertebral body, L1, L3, L4, L1–L4, L1–L5, the screw trajectory, the pedicle, the upper instrumented vertebra, adjacent vertebrae, and endplate-related regions (Table 1).

### 3.3 Overall association between preoperative CT HU and postoperative mechanical complications

The main continuous analysis included twelve studies that reported an adjusted odds ratio per Hounsfield unit. Each one-unit increase in preoperative HU was associated with lower odds of overall postoperative mechanical complications (OR 0.983; 95% CI, 0.978–0.989; *P* < 0.001). The corresponding 95% prediction interval was 0.974–0.993 per 1 HU (0.77–0.93 per 10 HU), lying below the null but spanning small to moderate effects. On a per-10-HU scale the pooled odds ratio was 0.846 (95% CI, 0.802–0.891) (Figure 2).

**Figure 2:**
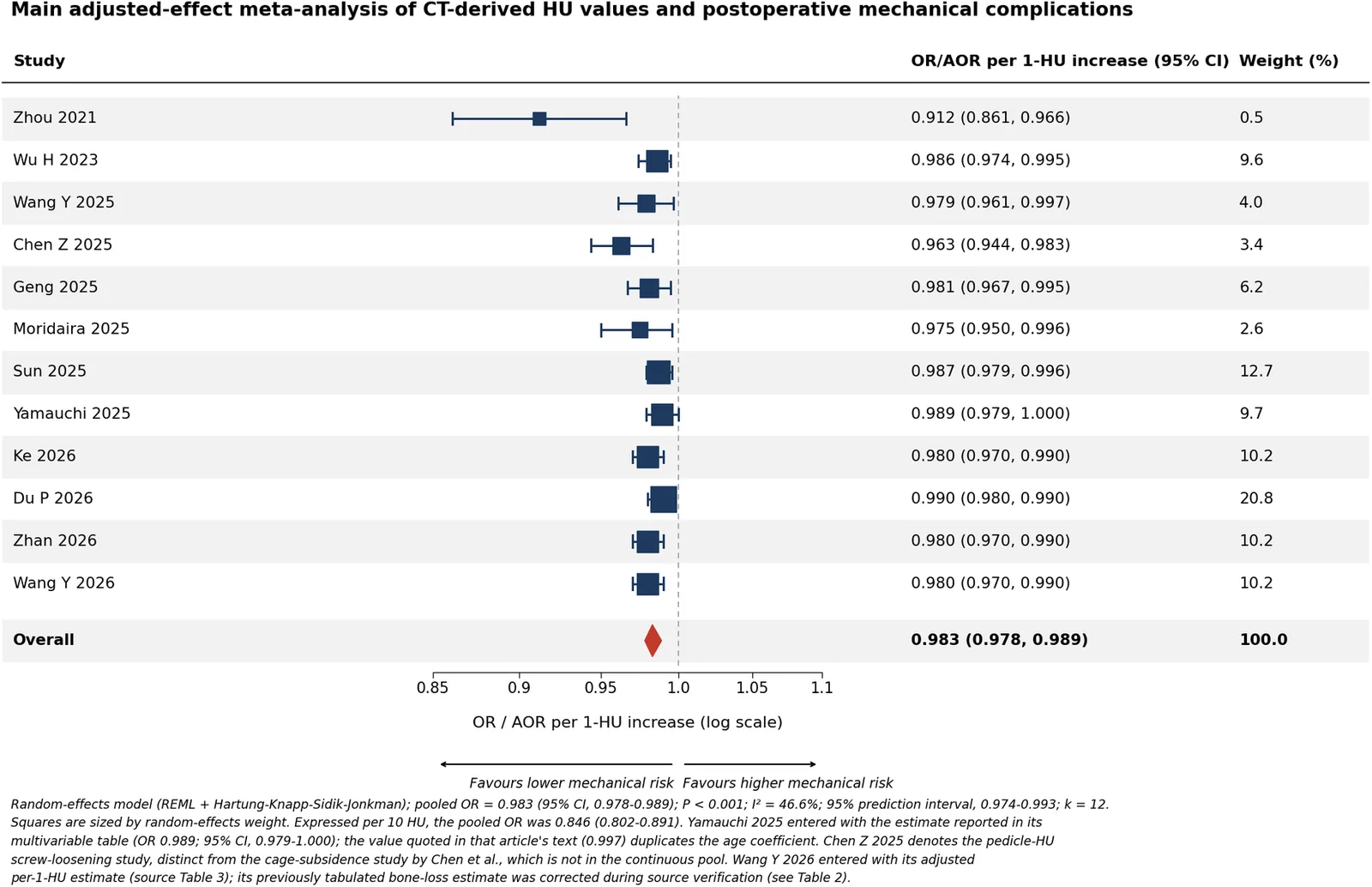
Main forest plot of the continuous per-hounsfield-unit association with postoperative mechanical complications (k = 12). Estimates are adjusted odds ratios expressed per 1-HU increase. Squares are study-specific estimates sized by inverse-variance weight; horizontal lines are 95% confidence intervals; the diamond is the random-effects pooled estimate (REML with Hartung-Knapp-Sidik-Jonkman intervals). The dashed vertical line marks the null (OR = 1). The pooled odds ratio was 0.983 (95% CI, 0.978–0.989; *P* < 0.001), with moderate heterogeneity (I^2^ = 46.6%) and a 95% prediction interval of 0.974–0.993; on a per-10-HU scale the pooled estimate was 0.846 (0.802–0.891). Yamauchi et al. (40) is entered with the estimate from its multivariable table (OR 0.989; 95% CI, 0.979–1.000). Chen et al. denotes the pedicle-HU screw-loosening study [reference (31); Table 1], distinct from Chen et al. [reference (30); cage subsidence], which is not in the continuous pool. Wang et al. (47) is entered with its adjusted per-HU estimate for preoperative HU.

Heterogeneity was moderate (I^2^ = 46.6%; *τ*^2^ = 0.000013; Q = 20.6, *P* = 0.038). Leave-one-out re-pooling left the estimate between 0.982 and 0.984; the largest reductions in heterogeneity followed omission of Du et al. (46), the most heavily weighted study (I^2^ = 30.9%), and of Zhou et al. (15), the only study at high risk of bias (I^2^ = 27.2%). When the model was restricted by covariate adjustment, the eight studies that adjusted for age gave a pooled odds ratio of 0.981 per 1 HU (95% CI, 0.971–0.991; *P* = 0.003) and the four that also adjusted for sex gave 0.987 (95% CI, 0.978–0.995; *P* = 0.016; 0.874 per 10 HU) (Supplementary Tables S9 and S10).

The extracted adjusted estimates were mainly derived from multivariable logistic regression or Cox regression models. Continuous HU estimates, dichotomous HU cut-off comparisons, and HR-based estimates were classified before synthesis rather than being pooled in a single model. The extracted estimates and their analysis categories are shown in Table 2.

**Table 2:** Extracted adjusted effect estimates and analysis categories.

Study	Outcome	Effect measure	Predictor scale (as reported)	Effect estimate (95% CI)	*P* value	Analysis assignment (Table S5)	Note
Zhou 2021 (15)	Cage subsidence	OR	Per 1 HU	0.912 (0.861–0.966)	0.002	Continuous only	
Gong 2022 (16)	Cage subsidence	AOR	L1 HU <110 vs ≥110	9.00 (1.30–64.20)	0.028	Binary only	g
Li 2022 (19)	Screw loosening	OR	Per 1 HU	0.95 (0.93–0.97)	<0.001	Not pooled †	
Xie 2022 (20)	Cage subsidence	OR	Per 1 HU	1.07 (1.04–1.09)	<0.001	Not pooled †	c
Zhang 2022 (21)	PJK	OR	Per 1 HU	0.98 (0.96–0.99)	0.002	Not pooled †	
Wu 2023 (23)	Intraoperative endplate violation	OR	Per 1 HU (endplate)	0.986 (0.974–0.995)	0.008	Continuous + binary	
Li 2023 (24)	Screw loosening	OR	Per 1 HU	0.94 (0.91–0.97)	<0.001	Not pooled †	
Yao 2023 (25)	CS/SL	AOR	L1 HU < 117 vs ≥ 117 (dichotomized)	4.10 (2.17–7.74)	<0.001	Binary only †	g
Zhao 2023 (26)	Screw loosening	OR	Per 1 HU	0.98 (0.97–0.99)	<0.001	Not pooled †	
Arimura 2024 (27)	Screw loosening	AOR	Per 1 unit of HU/pentosidine ratio	0.09 (0.02–0.53)	0.007	Directional only	k
Zhang 2024 (28)	Screw loosening	OR	Per 1 pedicle HU (screw-level; as reported)	1.012 (1.005–1.019)	<0.001	Binary only	d
Chen 2025 (30)	Cage subsidence	AOR	L1 CT-attenuation category (<110/110–160/ > 160 HU); per higher category	0.07 (0.02–0.24)	<0.001	Binary only †	g
Chen 2025 (31)	Screw loosening	AOR	Per 1 HU	0.963 (0.944–0.983)	<0.001	Continuous + binary	b
Ding 2025 (32)	Cage subsidence	AOR	Per 1 HU (L4 model; multiple regional models reported)	0.982 (0.968–0.996)	0.001	Binary only	i
Geng 2025 (33)	PJK/PJF	AOR	Per 1 HU	0.981 (0.967–0.995)	0.009	Continuous + binary	
Koo 2025 (34)	Cage subsidence	AOR	Per 1 HU (as reported; coding direction unresolved)	1.017 (1.004–1.031)	0.012	Binary only	h
Liu 2025 (35)	Cage subsidence	HR	Per 1 HU	0.97 (0.95–0.98)	<0.001	Binary only + HR	
Moridaira 2025 (36)	PJF	AOR	Per 1 HU	0.975 (0.950–0.996)	0.016	Continuous + binary	
Pennington 2025 (37)	PJK	HR (univariable Cox)	Per 1 HU (mean of UIV/UIV+1)	1.00 (0.99–1.01)	0.39	HR narrative	e
Pennington 2025 (37)	PJK	HR (univariable Cox)	HU <164 vs ≥164	3.05 (1.03–9.03)	0.04	HR narrative + directional	e
Sun 2025 (38)	Cage subsidence	AOR	Per 1 HU	0.987 (0.979–0.996)	0.003	Continuous only	
Yamamoto 2025 (39)	Screw loosening	AOR	Per 1 trajectory HU (screw-level)	0.98 (0.97–0.99)	0.002	Binary only †	j
Yamauchi 2025 (40)	Mechanical complications	AOR	Per 1 HU	0.989 (0.979–1.000)	0.046	Continuous + binary	a
Yuan 2025 (41)	Distal loosening	AOR	L1 HU <169 vs ≥169 (model coding inferred from methods)	10.67 (2.06–55.19)	0.005	Binary only †	g
Wang 2025 (42)	Cage subsidence	OR	Per 1 HU	0.979 (0.961–0.997)	0.022	Continuous + binary	
Levy 2026 (43)	Cage subsidence	OR	Endplate HU < 369 vs ≥ 369 (dichotomized)	5.70 (1.88–19.21)	<0.001	Binary only	g
Ke 2026 (44)	Screw loosening	AOR	Per 1 HU	0.980 (0.970–0.990)	<0.001	Continuous + binary	
Zhao 2026 (45)	Cage subsidence	OR	HU (scale not specified)	0.28 (0.12–0.57)	0.001	Not pooled †	g
Du 2026 (46)	Pedicle screw loosening	OR	Per 1 HU	0.990 (0.980–0.990)	<0.001	Continuous + binary	
Wang 2026 (47)	Cage subsidence	AOR	Per 1 HU (preoperative L1–L4 HU)	0.98 (0.97–0.99)	0.01	Continuous + directional	f
Yang 2026 (48)	Screw loosening	AOR	Per 0.1 unit of trajectory/vertebral HU ratio	0.19 (0.08–0.44)	<0.001	Descriptive only	
Zhan 2026 (49)	Cage subsidence	AOR	Per 1 HU	0.980 (0.970–0.990)	0.018	Continuous only	

Of the 35 included studies, the 31 shown here each provided a regression-based effect estimate; the remaining four [Lee et al. (17), Muheremu et al. (18), Shu et al. (29), and Hiyama et al. (22)] contributed only a crude low-versus-high contrast or group-mean comparison and are therefore summarized in the dichotomous synthesis (Figure 3 or, for directional-only evidence, Supplementary Table S5) rather than listed here. Estimates are shown exactly as reported by each study; the “Analysis assignment” column states which synthesis, if any, each estimate entered (Supplementary Table S5). For the studies displayed in Figure 3, the plotted odds ratio is either the study's reported odds ratio for the dichotomised HU variable or a crude 2 × 2 odds ratio reconstructed from source data (Supplementary Table S12), and may therefore differ from the regression estimate retained here. Footnotes: a, the source's multivariable table reports OR 0.989 (95% CI, 0.979–1.000; *P* = 0.046), whereas its text quotes 0.997, which duplicates the age coefficient; the tabulated estimate was used; b, age-adjusted pedicle-HU model, the estimate entered in the pooled analysis; c, as reported; direction inconsistent with the study's own group-level finding (lower HU in the subsidence group) and not reconcilable from the source, therefore not pooled; d, as reported; direction inconsistent with the study's own group-level finding and predictor scale unclear, therefore not entered into the continuous pool (its low-versus-high contrast is used in the dichotomous synthesis); e, two univariable Cox estimates reported by the same study; f, adjusted estimate for preoperative HU; the study's per-SD estimate for postoperative bone loss (OR 1.96) is not an HU effect and is not shown; g, dichotomized or categorical predictor as reported by the study, not harmonizable to a per-1-HU scale; h, as reported, but the authors' own interpretation implies the opposite direction, and the estimate was therefore not pooled; i, Ding et al. (32) reported multiple correlated regional models without a prespecified choice and was not continuously pooled; j, Yamamoto et al. (39) analysed 212 screws from 47 patients without a reported patient-clustered model and was not pooled with patient-level estimates; k, Arimura et al. (27) reported an HU-to-pentosidine ratio rather than HU alone and contributed directional evidence only. AOR, adjusted odds ratio; CI, confidence interval; CS, cage subsidence; HR, hazard ratio; HU, Hounsfield unit; ML, machine learning; OR, odds ratio; PJF, proximal junctional failure; PJK, proximal junctional kyphosis; SD, standard deviation; SL, screw loosening.

### 3.4 Outcome-specific analyses

Exploratory analyses stratified by complication type were constrained by the small number of studies reporting a per-Hounsfield-unit odds ratio within each category, three for screw loosening, five for cage subsidence, and two for junctional outcomes. Screw loosening showed a directionally consistent but imprecise estimate (k = 3; OR 0.980; 95% CI, 0.949–1.012; *P* = 0.117), cage subsidence a significant inverse association (k = 5; OR 0.982; 95% CI, 0.971–0.993; *P* = 0.010), and the two junctional studies were listed at study level without pooling [Geng et al. (33), OR 0.981, 95% CI 0.967–0.995; Moridaira et al. (36), OR 0.975, 95% CI 0.950–0.996]. These estimates are reported in Supplementary Table S8 and Supplementary Figure S1.

A second body of evidence compared groups defined as low or high HU by a study-specific threshold. Twenty-five studies furnished such a contrast, and every one placed the lower-HU group at higher risk of mechanical complications. The thresholds, however, were not comparable: they ranged from roughly 71–369 HU and were drawn at different vertebral sites, including L1, the L1–L4 mean, L4, the pedicle, S1, the sacrum, the screw trajectory, the endplate, and the upper instrumented vertebra. Because an odds ratio computed against a study-specific cut-off depends on that cut-off, and because the cut-offs and measurement sites were so disparate, these contrasts were not combined into a pooled estimate. Eleven studies provided an odds ratio that could be reported or reconstructed exactly from the source: four reported an odds ratio for the dichotomised HU variable [Gong et al. (16), Yao et al. (25), Yuan et al. (41), and Levy et al. (43); the latter two adjusted] and seven yielded a crude 2 × 2 odds ratio from reported counts or from integer-exact sensitivity and specificity (Supplementary Table S12). Their estimates ranged from about 2.3–58.7, all on the same side of unity (Figure 3); the most extreme value (31) rests on a single event in the high-HU group. The remaining fourteen studies supported the same direction through evidence that could not be expressed as such an odds ratio: two reported hazard ratios; three reported group-mean comparisons only (22, 29, 47); one reported an odds ratio for a Hounsfield-unit-to-pentosidine ratio rather than HU alone; and eight reported a threshold without group counts, without a confidence interval, with a three-level classification, or with sensitivity and specificity that do not resolve to integer counts [(18, 23, 28, 30, 33, 39, 40, 46); Supplementary Table S5].

**Figure 3:**
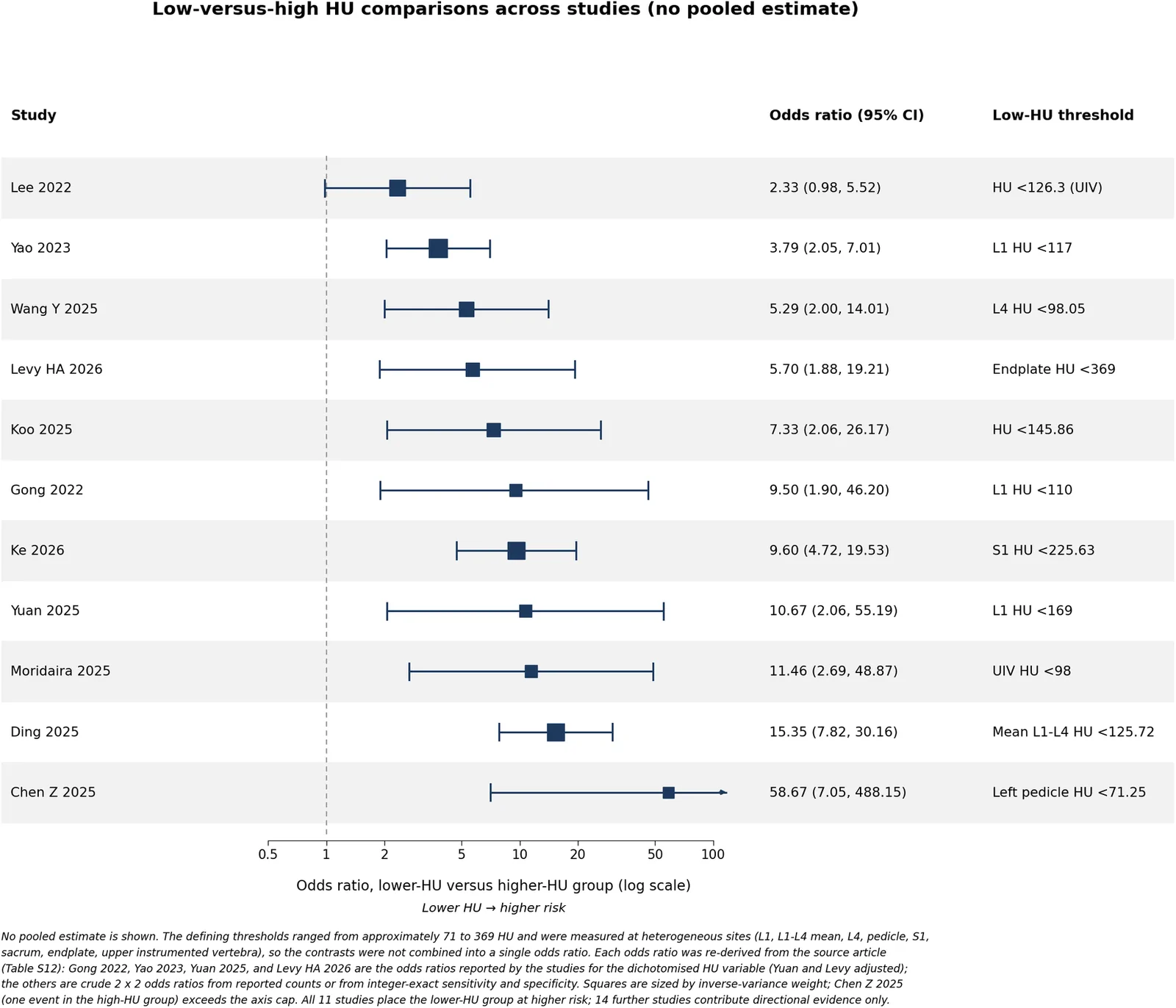
Low-versus-high HU comparisons, shown without a pooled estimate (k = 11). Gong et al. (16), Yao et al. (25), Yuan et al. (41), and Levy et al. (43) are the odds ratios reported by the studies for the dichotomised HU variable (Yuan and Levy adjusted); the others are crude 2 × 2 odds ratios reconstructed from reported counts (36) or from sensitivity and specificity that resolve exactly to integer event counts (Supplementary Table S12). Squares are sized by inverse-variance weight; horizontal lines are 95% confidence intervals. No summary diamond is shown: the defining thresholds ranged from approximately 71–369 HU and were drawn at incompatible anatomical sites, so a single pooled odds ratio would not estimate a consistent quantity. Study-level estimates ranged from about 2.3–58.7, all favoring higher risk in the lower-HU group; Chen et al. (31) (OR 58.67; 95% CI, 7.05–488.15; one event in the high-HU group) exceeds the axis cap and is shown with an arrow. Fourteen further studies supported the same direction through evidence that could not be expressed as such an odds ratio (Supplementary Table S5) and are not plotted.

### 3.5 Subgroup analyses

Prespecified subgroup analyses were performed within the main continuous analysis. Stratification by HU measurement site, surgical approach, and spinal region showed pooled estimates that were similar across subgroups, with no significant between-subgroup differences (Supplementary Table S1) (QM *P* = 0.89 for HU measurement site, 0.97 for surgical approach, and 0.65 for spinal region); subgroup-specific estimates were directionally consistent with the overall result. Because biomechanical differences between measurement sites are a specific concern, vertebral-body measurements (k = 8; OR 0.983; 95% CI, 0.977–0.988) were also compared directly with pedicle or screw-trajectory measurements (k = 2; OR 0.978; 95% CI, 0.822–1.164); no difference was detected (QM *P* = 0.61). With one or two studies in several subgroups, variable measurement protocols within categories (Supplementary Table S11), and no correction for multiple comparisons, these tests are underpowered and do not establish equivalence between sites. Outcome-specific subtotals for screw loosening and cage subsidence are reported in Supplementary Table S8 and Supplementary Figure S1. Within the low-versus-high comparisons, the direction of effect was consistent across subgroups defined by outcome category, measurement site, surgical approach, and spinal region.

### 3.6 Sensitivity analyses

Across random-effects estimators (REML, DerSimonian–Laird, and Paule–Mandel) the pooled odds ratio remained between 0.982 and 0.983, and a fixed-effect model gave 0.985 (Supplementary Table S2). Exclusion of the one study at high risk of bias gave 0.984 (95% CI, 0.980–0.988; Supplementary Table S3).

### 3.7 Publication bias and sensitivity analysis

The funnel plot for the main continuous analysis was asymmetric, with the less precise studies scattered away from the pooled estimate (Figure 4). Egger's regression test was significant (*P* < 0.001). Duval–Tweedie trim-and-fill (L0 estimator; side of imputation selected from the data) imputed five studies to the right of the pooled estimate and gave an adjusted OR of 0.986 (95% CI, 0.982–0.989; Supplementary Figure S2), leaving the direction and significance of the association unchanged. The low-versus-high contrasts were not pooled and differ in threshold, measurement site, outcome, and analysis unit; no funnel plot or small-study-effect test was applied to them. In the leave-one-out analysis, no single omission moved the pooled estimate beyond 0.982–0.984 or changed its significance (Figure 5).

**Figure 4:**
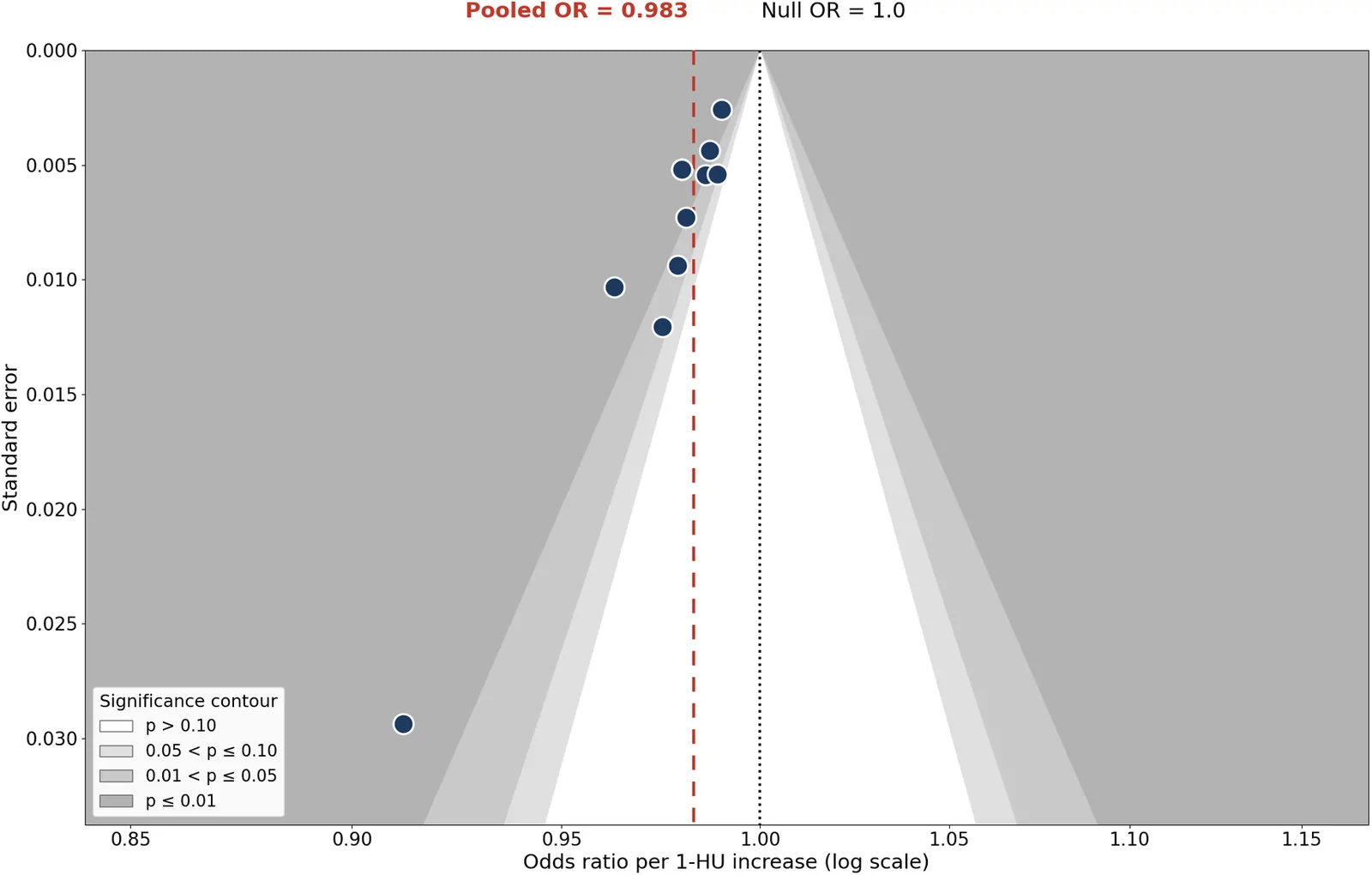
Contour-enhanced funnel plot for the continuous analysis (k = 12). The *x*-axis shows the OR per 1-HU increase on a log scale; the *y*-axis shows the standard error. The red dashed line marks the pooled estimate (OR = 0.983); the black dotted line marks the null (OR = 1). Asymmetry may reflect small-study effects, publication bias, or clinical and methodological heterogeneity.

**Figure 5:**
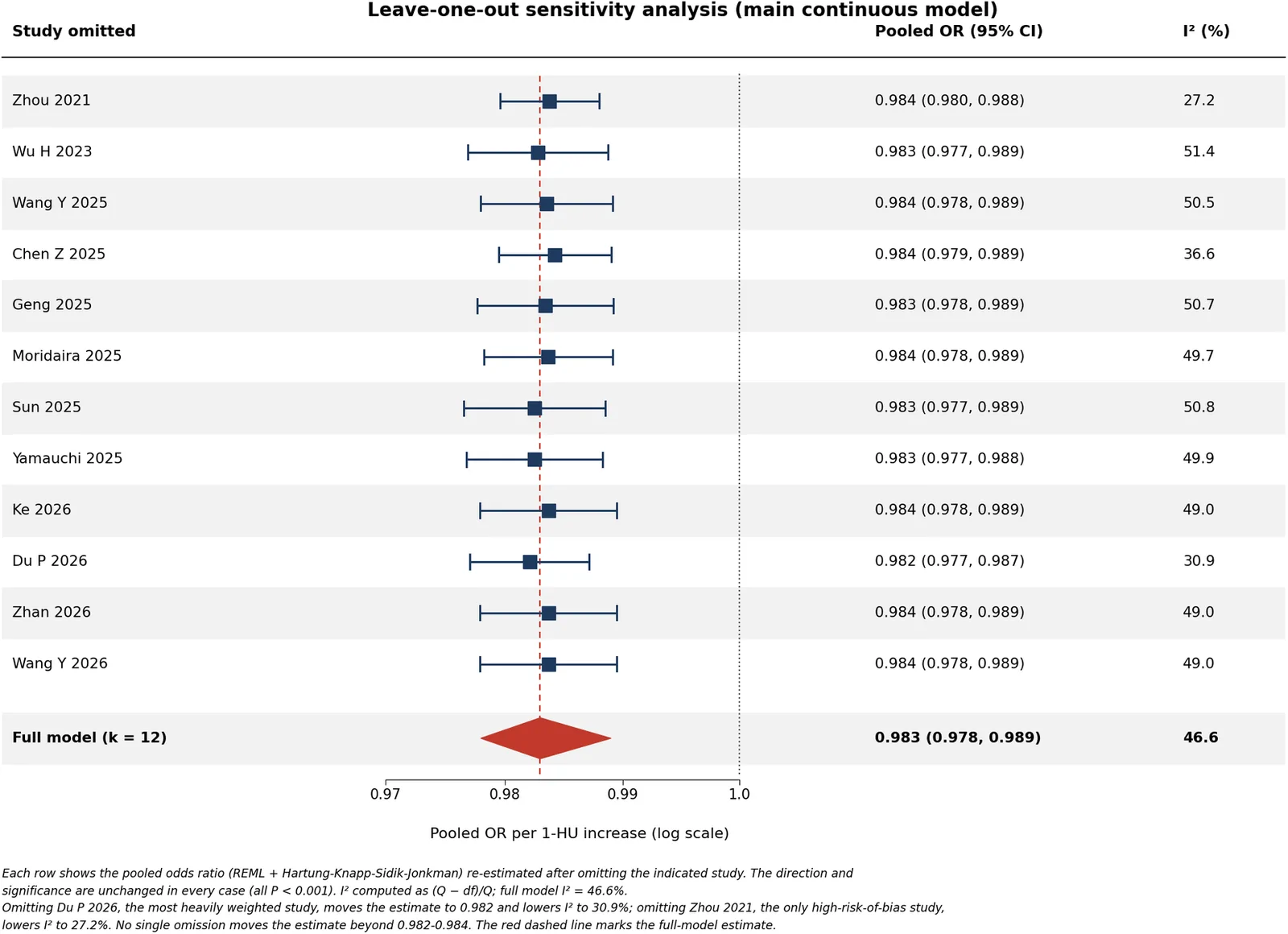
Leave-one-out sensitivity analysis for the continuous model (k = 11 per row). Each row shows the pooled OR (per 1-HU increase) re-estimated after omitting the indicated study. The red dashed line marks the full-data pooled estimate (OR = 0.983); the black dotted line marks the null (OR = 1). No single omission moved the estimate beyond 0.982–0.984 or altered its significance; omitting Du et al. (46) (the most heavily weighted study) or Zhou et al. (15) (the only high-risk-of-bias study) produced the largest reductions in heterogeneity (I^2^ 46.6%–30.9% and 27.2%, respectively).

### 3.8 Risk of bias assessment

Risk of bias was assessed using QUIPS-based domains (Figure 6). Each of the 35 included studies was assessed once at the study level. The heatmap showed that most studies were rated as low risk in study participation, study attrition, outcome measurement, and statistical analysis/reporting. Because all included studies were retrospective, confounding was treated as a key source of concern and was rated at least moderate. High-risk judgements were mainly concentrated in the prognostic factor measurement domain and the overall judgment (Figure 6A). Domain-level judgements for all 35 studies are reported in Supplementary Table S6.

**Figure 6:**
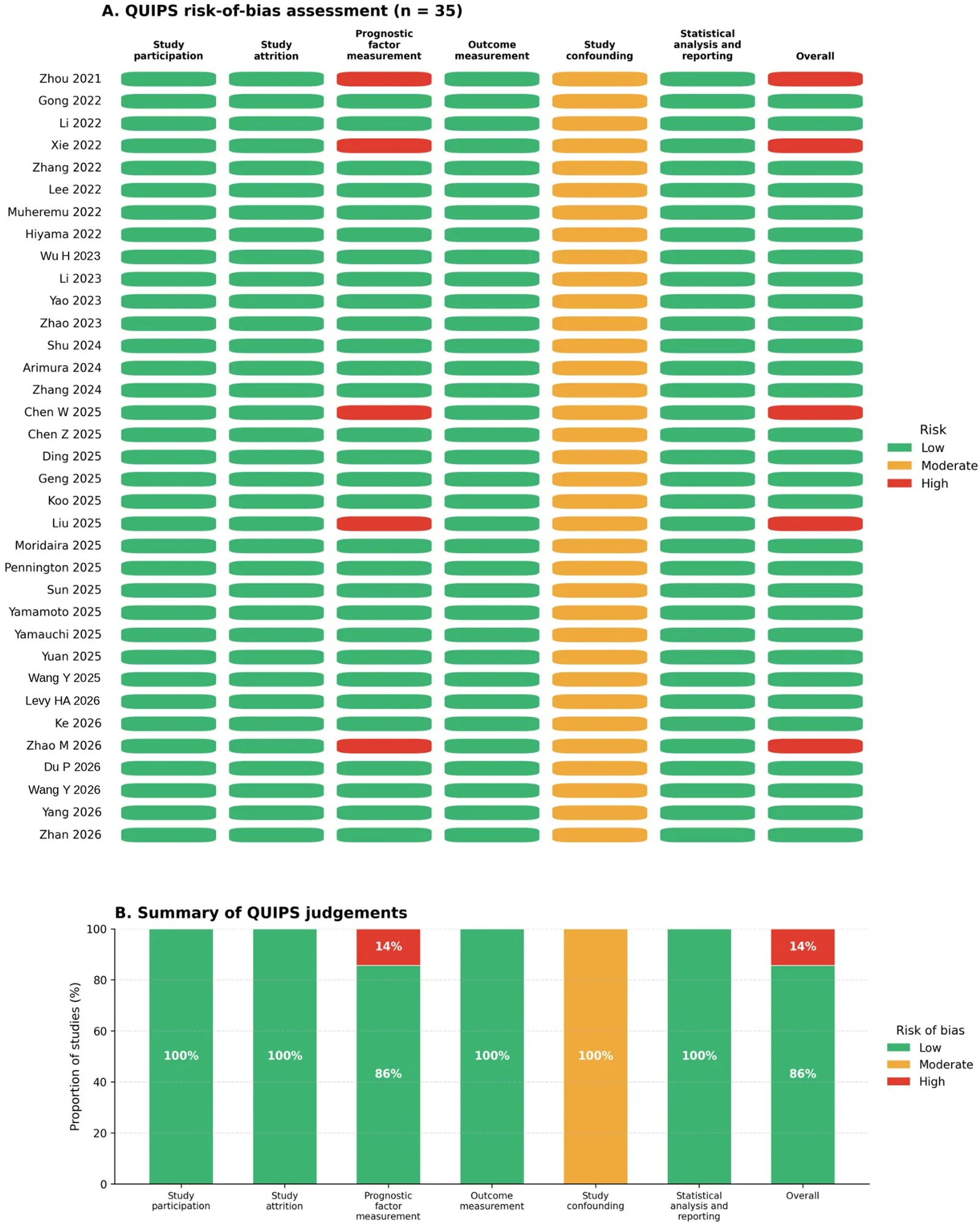
Risk-of-bias assessment using the quality in prognosis studies (QUIPS) tool. **(A)** Study-level risk-of-bias heatmap (*n* = 35). **(B)** Summary distribution across QUIPS domains.

In the summary plot, low-risk judgements predominated in most domains, and the confounding domain contributed the largest proportion of moderate-risk ratings, reflecting the retrospective design of all included studies (Figure 6B).

## 4 Discussion

Across thirty-five retrospective studies, the continuous per-Hounsfield-unit analysis showed a small, statistically significant inverse association between preoperative HU and postoperative mechanical complications, consistent in direction across every contributing study. The low-versus-high comparisons pointed the same way but rested on cohort-specific thresholds that could not be combined into a single estimate. Read together, the two scales describe one association at different resolutions rather than competing results: HU is an accessible marker of local bone quality whose per-unit effect is modest but directionally consistent.

The main continuous analysis showed an inverse and statistically significant association between preoperative HU and postoperative mechanical complications (OR 0.983 per HU; 95% CI, 0.978–0.989; *P* < 0.001). The estimate was stable across estimators, on leave-one-out re-pooling, on risk-of-bias-restricted re-pooling, and on removal of each outcome category in turn. The per-unit effect is small: each additional Hounsfield unit corresponds to roughly a 1.7% reduction in the odds of mechanical complications, and each 10 HU to about 15%. What remains uncertain is the magnitude of the association across clinical settings; the prediction interval lay below the null but spanned small to moderate effects (0.974–0.993 per HU), and all of the evidence is retrospective. HU is more appropriately read as one locally relevant marker within a preoperative risk assessment that also weighs age, comorbidity, DXA where available, and the number of instrumented segments.

Heterogeneity in the main analysis was moderate (I^2^ = 46.6%) and did not resolve into a single clinical source: subgroup analyses by HU measurement site, surgical approach, spinal region, and outcome category showed no between-subgroup differences, and meta-regression on publication year and follow-up duration identified no moderators. The residual dispersion is more plausibly methodological than clinical. The contributing studies measured HU at different vertebral sites with different region-of-interest rules, defined mechanical complications inconsistently, and adjusted for different covariate sets; some treated HU as a continuous variable and others as a threshold-based grouping, and a single L1 value may not represent the bone quality of the fused segments in patients with degeneration, deformity, or sclerosis. The heterogeneity concerned the magnitude of the effect rather than its direction, which was the same in all twelve studies. Vertebral-body, pedicle, screw-trajectory, endplate, and upper-instrumented-vertebra measurements sample biomechanically different bone and should not be treated as interchangeable, and the acquisition and measurement details reported by the included studies (Supplementary Table S11) show how variably they are currently obtained. Standardized measurement protocols that specify the CT acquisition (dedicated lumbar versus abdominopelvic examination, contrast phase, tube voltage, and slice thickness), the anatomical site, the plane and number of slices, and the rules for excluding cortical bone, the basivertebral venous plexus, and sclerotic lesions would allow estimates to be compared across centres and pooled with greater confidence; effect estimates should also be reported by outcome category and predictor scale.

The association did not concentrate in any one complication type. Lower HU has a plausible mechanism at the screw–bone interface: poor trabecular purchase permits micromotion, peri-screw radiolucency, and eventual loosening. A parallel rationale applies to cage subsidence, where endplate and subchondral bone resist settling, and to junctional failure, where the upper instrumented vertebra bears redistributed load. Yet when the continuous evidence was split by outcome, the screw-loosening subset was directionally consistent but imprecise (k = 3; confidence interval including the null), the cage-subsidence subset retained a significant inverse association (k = 5), and the junctional evidence was confined to two studies that were listed rather than pooled (Supplementary Table S8; Supplementary Figure S1). Removing screw-loosening, cage-subsidence, or junctional studies in turn left the pooled estimate significant and unchanged in direction (Supplementary Table S7). The composite estimate is a prespecified summary across mechanistically distinct events, not evidence that bone attenuation acts uniformly on each of them, and the outcome-specific estimates are the more appropriate basis for clinical interpretation.

The cage-subsidence estimate rests on five studies with moderate heterogeneity and differing subsidence definitions. Cage subsidence is multifactorial: endplate preparation, cage footprint, material, and position, restoration of segmental lordosis and disc height, and the surgical approach all influence load transfer across the disc space. For this outcome, HU is best interpreted alongside endplate morphology, cage characteristics, and surgical technique.

Evidence specific to proximal junctional kyphosis or failure was sparse. Only two studies reporting a per-HU estimate for junctional outcomes entered the continuous synthesis [Moridaira et al. (36), Geng et al. (33)], too few for a separate pooled analysis; their study-level estimates are listed in Supplementary Table S8 (Panel C) and Supplementary Figure S1. PJK and PJF arise from age, the magnitude of sagittal correction, the level of the upper instrumented vertebra and the integrity of its posterior soft tissues, rod stiffness, pelvic morphology, and perioperative strategy, of which bone attenuation is only one. Lower HU at the upper instrumented vertebra plausibly contributes to risk and is consistent with the overall inverse association, but the junctional data alone cannot support an HU-based threshold for these complications.

The low-versus-high contrasts cannot be separated from the thresholds used to create them. In nearly every study the cut-off was derived within the same cohort that supplied the outcome, most often by maximising the Youden index on a receiver-operating-characteristic curve; only one study used a value fixed in advance from the literature. A threshold chosen to best separate cases from non-cases in a sample widens the between-group contrast in that sample and pushes the odds ratio away from the null, as the two most extreme estimates illustrate [Chen et al. (31), OR 58.67 with a single event in the high-HU group; Ding et al. (32), OR 15.35]. The thresholds spanned roughly 71–369 HU at incompatible anatomical sites, so a single “low-HU” label denoted different anatomy and different absolute values from one report to the next, and a combined odds ratio would have averaged effects that do not estimate the same quantity. What the comparisons show consistently is direction: in every informative study the lower-HU group carried higher risk. The continuous model supplies the transportable estimate of magnitude, and the low-versus-high studies corroborate its direction. A universal HU threshold is unrealistic; outcome-specific and site-specific thresholds, each defined in one cohort and validated prospectively in another, are the priority for future work.

### Confounding is the principal threat to validity

All included studies were retrospective, and the adjustment variables differed between them: age, sex, body mass index, osteoporosis treatment, diabetes, smoking, sarcopenia, index level, screw dimensions, cage type, number of instrumented segments, alignment correction, and cement augmentation were not uniformly controlled. Concurrent anti-osteoporotic treatment was rarely reported and never adjusted for, although it could modify both HU and the risk of implant failure. Baseline bone mineral density by DXA entered only one contributing model (Supplementary Table S9), so HU and DXA-defined osteoporosis could not be disentangled. The number of instrumented segments, the magnitude of sagittal correction, and the surgical approach differed across cohorts and were inconsistently modelled; open and minimally invasive techniques alter load transfer at the screw–bone and cage–endplate interfaces and may change the risk of subsidence independently of bone quality, and the approach-stratified subgroups in Supplementary Table S1 were too small to separate these effects. Because HU is itself related to age, bone metabolism, degeneration, and deformity, the pooled association is a marker-level association observed within heterogeneous surgical contexts rather than an independent causal effect of bone attenuation.

The funnel plot was asymmetric and Egger's test was significant, but with twelve studies these findings cannot distinguish small-study effects or publication bias from clinical and methodological heterogeneity, and the less precise studies also differed in outcome definition and HU measurement. Trim-and-fill imputed five studies and shifted the estimate only marginally (adjusted OR 0.986). Small-study effects cannot be excluded.

The strengths of this review are its focus on a routinely available preoperative measurement, the separation of overall mechanical complications from specific outcomes, the separation of continuous adjusted estimates from dichotomous and hazard-ratio estimates rather than pooling across scales, and the use of a prognostic-study risk-of-bias framework.

### This review has several limitations

All included studies were retrospective, with the attendant risks of selection bias, information bias, and residual confounding; adjustment for sex and for bone mineral density was sparse (Supplementary Table S9), and the covariate-restricted analyses (Supplementary Table S10) rest on few studies. HU measurement sites and methods differed, and outcome definitions were not standardized, particularly for cage subsidence and overall mechanical complications; these methodological differences appear to be a larger source of heterogeneity than patient- or surgery-level factors, given the absence of moderators in subgroup and meta-regression analyses. Outcome-specific analyses, especially for PJK and PJF, included few studies. Because the included studies were rarely pre-registered, selective outcome reporting cannot be excluded, although the direction of association was consistent across the outcomes reported within studies (Supplementary Table S5). Most studies were conducted in East Asian populations, which may limit generalisability. Subgroup analyses were hypothesis-generating, with no correction for multiple comparisons. Finally, HU effect estimates are reported so heterogeneously that only 11 of the 25 low-versus-high contrasts could be reproduced exactly from their sources, which limits the descriptive synthesis and underlines the need for the standardized reporting recommended above.

Under a GRADE framework adapted for prognostic-factor reviews, certainty for the continuous analysis was low (Supplementary Table S4): the evidence began at low certainty because every study was observational, and it was not downgraded further, because the heterogeneity reflected methodological differences around a consistent direction of effect, the pooled association excluded the null and was stable on sensitivity analysis, and trim-and-fill moved the estimate only marginally; the judgement on publication bias carries uncertainty because Egger's test was significant. The low-versus-high comparison was not pooled and was not graded; it is carried as qualitative evidence of direction. Low certainty supports using HU as one element of a multifactorial preoperative risk assessment rather than as a standalone predictor or a fixed threshold.

### Clinical implications

Dual-energy x-ray absorptiometry is often unavailable at the time of surgical planning and is prone to artefactual elevation by lumbar degeneration, aortic calcification, and prior instrumentation, whereas a vertebral HU value is already present on every preoperative CT. A low value is an opportunistic, no-additional-cost signal that should prompt formal bone-health assessment and, where appropriate, medical optimisation; it is not a diagnostic endpoint in itself. Because the published thresholds were wide and cohort-specific (approximately 71–369 HU) and were mostly derived and evaluated within the same cohort, HU is best used as an opportunistic marker of possibly impaired bone quality that prompts further metabolic bone evaluation and individualized surgical planning, not as a validated categorical risk-stratification tool. Within an assessment that also weighs age, sagittal alignment, and the number of instrumented segments, each further decrement in HU corresponds to a continuous increase in estimated risk (a 10-HU decrease to roughly an 18% relative increase in the odds of mechanical complication) rather than to the crossing of a single cut-point. In patients with low HU, surgeons may consider established options for reinforcing fixation, such as cement-augmented pedicle screws, which reduced screw loosening in osteoporotic cohorts (odds ratio 0.18, 95% CI 0.09–0.38) (13), and cortical bone trajectory screws, which engage denser cortical bone and showed less loosening than the traditional trajectory in comparative series (14). That evidence derives from osteoporosis-defined, largely DXA-based populations rather than from HU-selected patients, and no study has shown that acting on a specific HU value reduces mechanical failure; the association reported here is not a treatment recommendation. The evidence also differs by outcome: the cage-subsidence subset retained a significant inverse association, the screw-loosening subset was directionally consistent but imprecise, and the data for proximal junctional kyphosis and failure were sparse.

The pooled per-unit estimate is not a bedside calculator; translating a single coefficient into an individual decision will require purpose-designed prospective studies. In the meantime, the most useful application of HU is comparative measurement at several sites within the same patient. Reading a global measure (lumbar DXA or an averaged lumbar HU) together with a local HU at the index level guards against the overestimation of areal density by lumbar degeneration (2), so that a patient with a reassuring DXA but a low local HU is still identified. Comparing HU within the instrumented vertebra, at the screw trajectory, the pedicle, and the subchondral bone beneath the endplate, informs the choice of fixation: a low pedicle or trajectory HU predicts screw loosening more strongly than whole-vertebral HU (26, 31, 39, 46) and supports consideration of cement-augmented or cortical bone trajectory screws, whereas a low endplate HU predicts endplate violation and cage subsidence (23) and supports a larger-footprint interbody cage or an approach that seats the cage on the denser apophyseal ring. Comparing HU across the vertebrae at the ends of a planned instrumentation, the upper instrumented and adjacent levels, informs the selection of fusion levels and of the upper instrumented vertebra, since low HU at these levels is associated with proximal junctional kyphosis or failure (33, 36). None of these uses depends on a universal threshold; each treats HU as a within-patient comparative signal, while its absolute per-unit calibration awaits prospective validation.

## 5 Conclusions

Lower preoperative CT-derived Hounsfield unit values are associated with a higher risk of postoperative mechanical complications after thoracolumbar or lumbar instrumented fusion, an association that was consistent in direction across studies and outcome categories but imprecise within individual complication types and preliminary for proximal junctional kyphosis or failure. The composite estimate is a prespecified summary across mechanistically distinct events. The HU value, available at no additional cost on routine preoperative CT, is best used as an opportunistic marker within individualized preoperative planning: a low value should prompt formal bone-health evaluation and closer surgical scrutiny, rather than serve as a validated threshold tool, a fixed per-unit predictor, or a trigger for specific interventions whose benefit in HU-selected patients remains unproven.

## Data Availability

The original contributions presented in the study are included in the article/Supplementary Material, further inquiries can be directed to the corresponding authors.
