# Supplementary material for: Preoperative CT-derived Hounsfield unit values and mechanical complications after thoracolumbar or lumbar instrumented fusion: a systematic review and meta-analysis
Source: Front Surg. 2026 Oct 7;13:1909831. doi: 10.3389/fsurg.2026.1909831 (PMC13684424; doi:10.3389/fsurg.2026.1909831)
Supplement: Supplementary file 1 [file Supplementaryfile1.docx]

**Supplementary Materials**

*Preoperative CT Hounsfield units and postoperative mechanical complications after thoracolumbar and lumbar fusion: a systematic review and meta-analysis*

**Supplementary Methods**

**Supplementary Methods S1. Full PubMed search strategy**

*("Hounsfield Unit" OR "Hounsfield units" OR HU OR "CT value" OR "computed tomography value" OR "computed tomography" OR CT)*

*AND*

*("spinal fusion" OR "lumbar fusion" OR "thoracolumbar fusion" OR "instrumented fusion" OR instrumentation OR "pedicle screw" OR "internal fixation")*

*AND*

*("screw loosening" OR "pedicle screw loosening" OR "cage subsidence" OR "proximal junctional kyphosis" OR PJK OR "proximal junctional failure" OR PJF OR "mechanical complication" OR "implant failure" OR pseudarthrosis OR nonunion OR revision)*

Search terms included combinations of the following keywords and Medical Subject Headings where applicable: "Hounsfield unit", "HU", "computed tomography", "CT value", "bone mineral density", "osteoporosis", "spinal fusion", "lumbar fusion", "thoracolumbar fusion", "instrumentation", "pedicle screw", "screw loosening", "cage subsidence", "proximal junctional kyphosis", "proximal junctional failure", "mechanical complication", "implant failure", "pseudarthrosis", and "revision surgery". The full search strategies for Embase, Web of Science, Cochrane Library, Scopus, and Google Scholar are available in the PROSPERO registration record (CRD420261376259).

**Table S1. Prespecified subgroup analyses of the main continuous adjusted-effect model.**

| **Moderator** | **Subgroup** | **k** | **OR** | **95% CI** | **P value** | **Q-between P** |
| --- | --- | --- | --- | --- | --- | --- |
| HU measurement site | Vertebral body | 8 | 0.983 | 0.977–0.988 | <0.001 | 0.89 |
|  | Pedicle/trajectory | 2 | 0.978 | 0.822–1.164 | 0.354 |  |
|  | Endplate | 1 | 0.986 | 0.974–0.995 | — |  |
|  | UIV/adjacent | 1 | 0.975 | 0.950–0.996 | — |  |
| Surgical approach | PLIF/TLIF | 7 | 0.983 | 0.976–0.989 | <0.001 | 0.97 |
|  | Posterior | 3 | 0.985 | 0.970–1.000 | 0.053 |  |
|  | OLIF | 2 | 0.953 | 0.583–1.558 | 0.433 |  |
| Spinal region | Lumbar/lumbosacral | 10 | 0.982 | 0.976–0.989 | <0.001 | 0.65 |
|  | Thoracolumbar/thoracic | 2 | 0.986 | 0.939–1.036 | 0.173 |  |

Subgroup-specific estimates are random-effects pooled odds ratios per 1-Hounsfield-unit increase (REML with Hartung–Knapp–Sidik–Jonkman intervals); single-study subgroups are shown as the individual study estimate and were not pooled (P not estimable). Between-subgroup P values are from the omnibus test of moderators (QM) of a mixed-effects meta-regression with a common between-study variance (REML with Hartung–Knapp–Sidik–Jonkman adjustment), computed across all subgroups of the moderator; a direct comparison of the vertebral-body (k = 8) and pedicle/screw-trajectory (k = 2) subgroups gave QM P = 0.61. Outcome-category subgroups are reported separately in Table S8. With 12 studies these analyses are exploratory and underpowered.

**Table S2. Estimator-comparison sensitivity analysis (main continuous model).**

| **Model** | **k** | **OR** | **95% CI** | **P value** | **τ²** |
| --- | --- | --- | --- | --- | --- |
| **REML + HKSJ (main)** | **12** | **0.983** | **0.978–0.989** | **<0.001** | **0.000013** |
| REML + Wald (z) | 12 | 0.983 | 0.980–0.987 | <0.001 | 0.000013 |
| DerSimonian–Laird | 12 | 0.983 | 0.978–0.987 | <0.001 | 0.000027 |
| Fixed-effect | 12 | 0.985 | 0.982–0.988 | <0.001 | — |

All estimators were applied to the same 12 effect estimates (one adjusted odds ratio per 1-Hounsfield-unit increase per study). REML, restricted maximum likelihood; HKSJ, Hartung–Knapp–Sidik–Jonkman adjustment (t-distribution, k − 1 degrees of freedom). The HKSJ interval used in the primary analysis is wider than the Wald-type interval and is reported as the main result; the inverse association is statistically significant under every estimator. The fixed-effect estimate is slightly closer to the null because it assigns larger inverse-variance weight to the most precise studies (Du P 2026, Sun 2025); it assumes a common true effect and therefore has no τ² estimate. The 95% prediction interval for the main random-effects model (REML + HKSJ; t-distribution with k − 2 degrees of freedom) was 0.974 to 0.993 per 1 HU (0.77 to 0.93 per 10 HU); it lay below the null but spanned small to moderate effects, reflecting moderate between-study heterogeneity (τ² = 0.000013; I² = 46.6%, calculated as (Q − df)/Q).

**Table S3. Risk-of-bias stratified sensitivity analysis (main continuous model).**

| **Analysis** | **k** | **OR** | **95% CI** | **P value** | **I²** |
| --- | --- | --- | --- | --- | --- |
| **Continuous, all studies** | **12** | **0.983** | **0.978–0.989** | **<0.001** | **46.6%** |
| Continuous, excluding high risk of bias | 11 | 0.984 | 0.980–0.988 | <0.001 | 27.2% |

Studies were classified by their overall QUIPS judgement (Table S6). Among the 12 studies in the main continuous model, one (Zhou 2021) was rated at high overall risk of bias; the second row re-pools after excluding it. Estimates are pooled adjusted odds ratios per 1-Hounsfield-unit increase (random-effects REML with Hartung–Knapp–Sidik–Jonkman intervals). The inverse association and its significance were unchanged. The secondary low-versus-high comparison was not pooled and is therefore not included in this stratified analysis.

**Table S4. GRADE certainty of evidence.**

| **Outcome** | **k** | **Risk of bias** | **Inconsistency** | **Indirectness** | **Imprecision** | **Publication bias** | **Certainty** |
| --- | --- | --- | --- | --- | --- | --- | --- |
| Overall mechanical complications (continuous, adjusted, per 1 HU) | 12 | Not serious (11/12 rated low overall risk, one high; stable on RoB-restricted re-pooling) | Not serious (moderate heterogeneity, I² = 46.6%, judged methodological; direction consistent in all 12 studies; prediction interval 0.974–0.993) | Not serious | Not serious (CI excludes null) | Not serious (asymmetry more plausibly heterogeneity; trim-and-fill imputed five studies with adjusted OR 0.986; Egger P < 0.001 but unreliable with k = 12) | **Low** |
| Low- vs high-HU comparison (binary, across outcomes) | 25 | — | — | — | — | — | **Not graded** |

Certainty was assessed with the GRADE framework adapted for prognostic-factor reviews (Huguet 2013; Iorio 2015). Bodies of observational prognostic evidence begin at low certainty. The continuous body began at low and was not downgraded further: the heterogeneity was judged to reflect methodological differences in HU measurement site and outcome definition rather than divergent true effects, the pooled association excluded the null and was robust to leave-one-out and risk-of-bias-restricted re-pooling (Tables S3, S7), and although the funnel plot showed visible asymmetry, in this small set of studies that asymmetry more plausibly reflected methodological heterogeneity than publication bias, and trim-and-fill moved the estimate only marginally (adjusted OR 0.986), so certainty was not downgraded further on this basis, although Egger's test was significant and this judgement carries uncertainty. The secondary low-versus-high comparison was not combined into a summary estimate and was therefore not formally graded; it is carried as qualitative, directionally consistent evidence. Outcome-specific subgroups (Tables S1, S8) were exploratory and underpowered and were not separately graded.

**Table S5. Study-by-analysis attribution matrix for the 35 included studies.**

| **Study** | **Ref** | **Continuous (per 1 HU)** | **Low- vs high-HU** | **HR** | **Pooled-analysis status** |
| --- | --- | --- | --- | --- | --- |
| **Liu 2025** | (35) | – | ✓ | ✓ | Binary only + HR |
| **Zhou 2021** | (15) | ✓ | – | – | Continuous only |
| **Gong 2022** | (16) | – | ✓ | – | Binary only |
| **Li 2022** | (19) | – | – | – | Not pooled † |
| **Xie 2022** | (20) | – | – | – | Not pooled † |
| **Zhang 2022** | (21) | – | – | – | Not pooled † |
| **Wu H 2023** | (23) | ✓ | ✓ | – | Continuous + directional ‡ |
| **Li 2023** | (24) | – | – | – | Not pooled † |
| **Yao 2023** | (25) | – | ✓ | – | Binary only † |
| **Zhao 2023** | (26) | – | – | – | Not pooled † |
| **Arimura 2024** | (27) | – | ✓ | – | Binary only |
| **Zhang 2024** | (28) | – | ✓ | – | Directional ‡ |
| **Chen W 2025** | (30) | – | ✓ | – | Directional ‡ |
| **Chen Z 2025** | (31) | ✓ | ✓ | – | Continuous + binary |
| **Ding 2025** | (32) | – | ✓ | – | Binary only |
| **Geng 2025** | (33) | ✓ | ✓ | – | Continuous + directional ‡ |
| **Koo 2025** | (34) | – | ✓ | – | Binary only † |
| **Moridaira 2025** | (36) | ✓ | ✓ | – | Continuous + binary |
| **Pennington 2025** | (37) | – | ✓ | ✓ | Binary only + HR |
| **Sun 2025** | (38) | ✓ | – | – | Continuous only |
| **Yamamoto 2025** | (39) | – | ✓ | – | Directional ‡ |
| **Yamauchi 2025** | (40) | ✓ | ✓ | – | Continuous + directional ‡ |
| **Yuan 2025** | (41) | – | ✓ | – | Binary only † |
| **Wang Y 2025** | (42) | ✓ | ✓ | – | Continuous + binary |
| **Levy HA 2026** | (43) | – | ✓ | – | Binary only |
| **Ke 2026** | (44) | ✓ | ✓ | – | Continuous + binary |
| **Zhao M 2026** | (45) | – | – | – | Not pooled † |
| **Du P 2026** | (46) | ✓ | ✓ | – | Continuous + directional ‡ |
| **Wang Y 2026** | (47) | ✓ | ✓ | – | Continuous + directional |
| **Yang 2026** | (48) | – | – | – | Descriptive only |
| **Zhan 2026** | (49) | ✓ | – | – | Continuous only |
| **Lee 2022** | (17) | – | ✓ | – | Binary only |
| **Muheremu 2022** | (18) | – | ✓ | – | Directional ‡ |
| **Shu 2024** | (29) | – | ✓ | – | Directional (mean-only) † |
| **Hiyama 2022** | (22) | – | ✓ | – | Directional (mean-only) † |

A check mark denotes contribution to that pooled analysis; reference numbers match the main reference list. The continuous model was restricted to the 12 studies reporting an adjusted odds ratio per 1 raw Hounsfield-unit increase. † The adjusted continuous estimate was reported on a non-unit scale (e.g., per 10 HU, per standard deviation, or grouped) and could not be placed on the per-unit continuous scale; such studies appear under the low-versus-high comparison where they reported a dichotomous contrast, otherwise they entered no pooled model. The low-versus-high comparison was not combined into a summary estimate; of the studies marked, 11 yielded an odds ratio reported by the study or reconstructed exactly from its data (Figure 3; derivation in Table S12) and 14 contributed directional support only (Liu 2025 and Pennington 2025 via Cox hazard ratios; Hiyama 2022, Shu 2024, and Wang Y 2026 via group-mean comparisons without a reproducible threshold-based 2×2 contrast; Arimura 2024 via a Hounsfield-unit-to-pentosidine ratio; and, marked ‡, eight studies whose threshold-level data could not be reconstructed exactly from the source: Geng 2025 reported no HU threshold, Chen W 2025 a three-level classification, Yamamoto 2025 a threshold odds ratio without a confidence interval, Zhang 2024 no counts by HU group, and the sensitivity/specificity of Yamauchi 2025, Wu H 2023, Muheremu 2022, and Du P 2026 do not resolve exactly to integer event counts). Shu 2023 and Shu 2024 report overlapping cohorts from one centre and are treated as a single study represented by the later and larger report. Liu 2025 and Pennington 2025 additionally reported Cox hazard ratios, handled narratively. Chen W 2025 and Chen Z 2025 are separate reports from different author groups. Yang 2026 met eligibility (Table 1) but could not be placed on either pooled scale.

**Table S6. Risk-of-bias assessment of the 35 included studies (QUIPS tool).**

| **Study** | **Ref** | **Participation** | **Attrition** | **Prognostic factor measurement** | **Outcome measurement** | **Confounding** | **Statistical analysis & reporting** | **Overall** |
| --- | --- | --- | --- | --- | --- | --- | --- | --- |
| **Zhou 2021** | (15) | Low | Low | High | Low | Moderate | Low | High |
| **Gong 2022** | (16) | Low | Low | Low | Low | Moderate | Low | Low |
| **Li 2022** | (19) | Low | Low | Low | Low | Moderate | Low | Low |
| **Xie 2022** | (20) | Low | Low | High | Low | Moderate | Low | High |
| **Zhang 2022** | (21) | Low | Low | Low | Low | Moderate | Low | Low |
| **Lee 2022** | (17) | Low | Low | Low | Low | Moderate | Low | Low |
| **Muheremu 2022** | (18) | Low | Low | Low | Low | Moderate | Low | Low |
| **Hiyama 2022** | (22) | Low | Low | Low | Low | Moderate | Low | Low |
| **Wu H 2023** | (23) | Low | Low | Low | Low | Moderate | Low | Low |
| **Li 2023** | (24) | Low | Low | Low | Low | Moderate | Low | Low |
| **Yao 2023** | (25) | Low | Low | Low | Low | Moderate | Low | Low |
| **Zhao 2023** | (26) | Low | Low | Low | Low | Moderate | Low | Low |
| **Shu 2024** | (29) | Low | Low | Low | Low | Moderate | Low | Low |
| **Arimura 2024** | (27) | Low | Low | Low | Low | Moderate | Low | Low |
| **Zhang 2024** | (28) | Low | Low | Low | Low | Moderate | Low | Low |
| **Chen W 2025** | (30) | Low | Low | High | Low | Moderate | Low | High |
| **Chen Z 2025** | (31) | Low | Low | Low | Low | Moderate | Low | Low |
| **Ding 2025** | (32) | Low | Low | Low | Low | Moderate | Low | Low |
| **Geng 2025** | (33) | Low | Low | Low | Low | Moderate | Low | Low |
| **Koo 2025** | (34) | Low | Low | Low | Low | Moderate | Low | Low |
| **Liu 2025** | (35) | Low | Low | High | Low | Moderate | Low | High |
| **Moridaira 2025** | (36) | Low | Low | Low | Low | Moderate | Low | Low |
| **Pennington 2025** | (37) | Low | Low | Low | Low | Moderate | Low | Low |
| **Sun 2025** | (38) | Low | Low | Low | Low | Moderate | Low | Low |
| **Yamamoto 2025** | (39) | Low | Low | Low | Low | Moderate | Low | Low |
| **Yamauchi 2025** | (40) | Low | Low | Low | Low | Moderate | Low | Low |
| **Yuan 2025** | (41) | Low | Low | Low | Low | Moderate | Low | Low |
| **Wang Y 2025** | (42) | Low | Low | Low | Low | Moderate | Low | Low |
| **Levy HA 2026** | (43) | Low | Low | Low | Low | Moderate | Low | Low |
| **Ke 2026** | (44) | Low | Low | Low | Low | Moderate | Low | Low |
| **Zhao M 2026** | (45) | Low | Low | High | Low | Moderate | Low | High |
| **Du P 2026** | (46) | Low | Low | Low | Low | Moderate | Low | Low |
| **Wang Y 2026** | (47) | Low | Low | Low | Low | Moderate | Low | Low |
| **Yang 2026** | (48) | Low | Low | Low | Low | Moderate | Low | Low |
| **Zhan 2026** | (49) | Low | Low | Low | Low | Moderate | Low | Low |

*QUIPS, Quality In Prognosis Studies. Each domain rated Low, Moderate, or High risk of bias. The study confounding domain was rated Moderate for all 35 studies, reflecting the retrospective design of every included cohort and the consequent potential for residual confounding. The prognostic factor measurement domain was rated High for five studies (Zhou 2021, Xie 2022, Chen W 2025, Liu 2025, Zhao M 2026) in which the Hounsfield-unit measurement was non-standardised, used a grouped or rescaled attenuation variable, or had insufficiently specified region-of-interest rules; the overall judgement for these five studies was High. All remaining domains and studies were rated Low risk. Overall, 30 of 35 studies (86%) were judged at low overall risk of bias and 5 (14%) at high overall risk.*

**Table S7. Leave-one-outcome-out sensitivity analysis of the main continuous model.**

| **Analysis** | **Studies (k)** | **Pooled OR (95% CI)** | **P value** | **I² (%)** |
| --- | --- | --- | --- | --- |
| **Full model (all outcomes)** | **12** | **0.983 (0.978–0.989)** | **<0.001** | **46.6** |
| Excluding screw-loosening studies | 9 | 0.983 (0.978–0.989) | <0.001 | 22.7 |
| Excluding cage-subsidence studies | 7 | 0.984 (0.977–0.991) | 0.002 | 43.8 |
| Excluding junctional (PJK/PJF) studies | 10 | 0.984 (0.978–0.990) | <0.001 | 54.0 |

Each row shows the pooled adjusted odds ratio per 1-Hounsfield-unit increase (random-effects REML model with Hartung–Knapp–Sidik–Jonkman confidence intervals; t-distribution with k − 1 degrees of freedom) re-estimated after removing all studies of one complication category. Removing the screw-loosening, cage-subsidence, or junctional (proximal junctional kyphosis/failure) studies in turn left the pooled estimate below unity and statistically significant in every case, indicating that the overall continuous association did not depend on any single complication type. The endplate-violation and mixed-mechanical categories each contained a single study and are therefore captured by the study-level leave-one-out analysis (Figure 5) rather than as separate rows. Between-study heterogeneity was low to moderate across all scenarios (I² 22.7% to 54.0%).

**Table S8. Exploratory outcome-specific subgroup analyses (continuous per-Hounsfield-unit model).**

| **Study** | **OR per 1-HU increase (95% CI)** | **Weight (%)** |
| --- | --- | --- |
| **A. Pedicle screw loosening (k = 3)** | | |
| Du P 2026 | 0.99 (0.98–0.99) | 41.4 |
| Chen Z 2025 | 0.963 (0.944–0.983) | 23.0 |
| Ke 2026 | 0.980 (0.970–0.990) | 35.6 |
| **Subtotal** | **0.980 (0.949–1.012); P = 0.117; I² = 77.6%** | **100.0** |
| **B. Cage subsidence (k = 5)** | | |
| Wang Y 2025 | 0.979 (0.961–0.997) | 8.7 |
| Zhou 2021 | 0.912 (0.861–0.966) | 0.9 |
| Sun 2025 | 0.987 (0.979–0.996) | 36.7 |
| Zhan 2026 | 0.980 (0.970–0.990) | 26.9 |
| Wang Y 2026 | 0.980 (0.970–0.990) | 26.9 |
| **Subtotal** | **0.982 (0.971–0.993); P = 0.010; I² = 50.6%** | **100.0** |
| **C. Proximal junctional kyphosis/failure (k = 2; study-level, not pooled)** |  |  |
| Geng 2025 | 0.981 (0.967–0.995) | — |
| Moridaira 2025 | 0.975 (0.950–0.996) | — |

Estimates are adjusted odds ratios per 1-Hounsfield-unit increase, pooled with a random-effects model (REML) using Hartung–Knapp–Sidik–Jonkman confidence intervals (t-distribution with k − 1 degrees of freedom); weights are REML random-effects weights. These analyses are exploratory: each subset contains few studies with moderate-to-high heterogeneity, and they are reported to show that the overall continuous association did not rest on any single complication type. The screw-loosening subtotal crosses the null (P = 0.117), whereas the cage-subsidence subtotal (k = 5) is statistically significant (P = 0.010); neither subset alone supports an outcome-specific Hounsfield-unit threshold. Panel C lists the two studies reporting an adjusted per-HU estimate for proximal junctional kyphosis or failure at study level; because they assessed biomechanically different sites (lumbar vertebrae versus the upper instrumented vertebra) and constructs, no pooled estimate was computed, and this evidence is regarded as preliminary. Du P 2026 reported its estimate to two decimal places, so its upper confidence limit coincides with the point estimate. Corresponding forest plots are shown in Supplementary Figure S1.

**Table S9. Covariate adjustment in the 12 studies contributing to the continuous per-Hounsfield-unit model.**

| **Study** | **PMID** | **Outcome (procedure)** | **Adjusted OR per 1 HU (95% CI)** | **Covariates entered in the multivariable model** | **Age** | **Sex** | **DXA / T-score BMD** |
| --- | --- | --- | --- | --- | --- | --- | --- |
| 1. Zhou 2021 | 34789220 | Cage subsidence (SA-OLIF) | 0.912 (0.861-0.966) | Age; L1-L4 HU; lowest T-score | ✓ | – | ✓ |
| 2. Wu H 2023 | 34736351 | Intraoperative endplate violation (OLIF) | 0.986 (0.974-0.995) | Regional endplate HU; no. of surgical levels; disc-height restoration (full set not enumerated) | NR | NR | – |
| 3. Wang Y 2025 | 39397676 | Cage subsidence (PLIF) | 0.979 (0.961-0.997) | Sex; age; BMI; fusion level; follow-up period | ✓ | ✓ | – |
| 4. Chen Z 2025 | 39575539 | Screw loosening (pedicle HU) | 0.963 (0.944-0.983) | Age (each HU index entered separately with age) | ✓ | – | – |
| 5. Geng 2025 | 40785926 | PJK/PJF (ADS) | 0.981 (0.967-0.995) | VBQ; preoperative SVA; postoperative SVA | – | – | – |
| 6. Moridaira 2025 | 40259287 | PJF (limited lumbar fusion) | 0.975 (0.950-0.996) | Age; PT; PI-LL; SVA (n=46) | ✓ | – | – |
| 7. Sun 2025 | 40331716 | Cage subsidence (PLIF) | 0.987 (0.979-0.996) | Sex; diabetes; PMI; MMI; FI; VBQ; EBQ; cage position/height; post-IH; corrected-IH; post-SA; corrected-SA (age not entered) | – | ✓ | – |
| 8. Yamauchi 2025 | 40566011 | Mechanical complications (ASD) | 0.989 (0.979-1.000) | Age; sex; SVA | ✓ | ✓ | – |
| 9. Ke 2026 | 41656211 | Screw loosening (PLIF) | 0.980 (0.970-0.990) | LIV at S1; L1 HU; S1 HU; L1-L4 VBQ; S1 VBQ | – | – | – |
| 10. Du P 2026 | 40879064 | Screw loosening (PLIF) | 0.990 (0.980-0.990) | Age; sex; BMI; number of fusion levels; LIV at S1 | ✓ | ✓ | – |
| 11. Zhan 2026 | 41788733 | Cage subsidence (single-segment LIF) | 0.980 (0.970-0.990) | Age; hypertension; VBQ; PBQ | ✓ | – | – |
| 12. Wang Y 2026 | 41904479 | Cage subsidence (PLIF) | 0.98 (0.97-0.99) | Sex; age; BMI; paravertebral muscle HU; postoperative vertebral bone loss; cage height | ✓ | ✓ | – |

*Each study's adjusted odds ratio per 1-HU increase was taken from the multivariable model listed. Adjustment sets were verified against the full text of each source study (PubMed Central). NR, not reported. VBQ/PBQ/EBQ, MRI-based vertebral/pedicle/endplate bone-quality scores (not DXA). Summary: age adjusted in 8/12, sex in 5/12, DXA/T-score BMD in 1/12 (Zhou 2021); age and sex jointly in 4/12; age, sex, and BMD jointly in 0/12. For Yamauchi 2025 the multivariable table reports OR 0.989 (95% CI, 0.979-1.000; SE 0.005; P = 0.046), whereas the text quotes 0.997, which is the age coefficient of the same table; the tabulated estimate was used.*

**Table S10. Sensitivity analyses of the continuous per-HU model restricted by covariate adjustment.**

| **Panel A — restricted to studies adjusting for age and sex (k = 4)** |  |  |
| --- | --- | --- |
| Study | OR per 1 HU (95% CI) | Weight (%) |
| Wang Y 2025 | 0.979 (0.961-0.997) | 8.5 |
| Yamauchi 2025 | 0.989 (0.979-1.000) | 21.0 |
| Du P 2026 | 0.990 (0.980-0.990) | 48.3 |
| Wang Y 2026 | 0.980 (0.970-0.990) | 22.2 |
| Subtotal — 0.987 (0.978-0.995); P = 0.016; I² = 25.3% |  | 100.0 |
| Panel B — restricted to studies adjusting for age (k = 8) |  |  |
| Study | OR per 1 HU (95% CI) | Weight (%) |
| Zhou 2021 | 0.912 (0.861-0.966) | 1.3 |
| Chen Z 2025 | 0.963 (0.944-0.983) | 8.0 |
| Moridaira 2025 | 0.975 (0.950-0.996) | 6.4 |
| Wang Y 2025 | 0.979 (0.961-0.997) | 9.2 |
| Zhan 2026 | 0.980 (0.970-0.990) | 17.1 |
| Yamauchi 2025 | 0.989 (0.979-1.000) | 16.6 |
| Du P 2026 | 0.990 (0.980-0.990) | 24.2 |
| Wang Y 2026 | 0.980 (0.970-0.990) | 17.1 |
| Subtotal — 0.981 (0.971-0.991); P = 0.003; I² = 63.3% |  | 100.0 |

*Adjusted odds ratios per 1-HU increase, pooled with the primary model (random-effects REML with Hartung-Knapp-Sidik-Jonkman intervals; t-distribution, k − 1 df). Weights are REML random-effects weights. Per-10-HU subtotals: Panel A, 0.874 (0.802-0.953); Panel B, 0.827 (0.746-0.916). I² calculated as (Q − df)/Q. Yamauchi 2025 is entered with the estimate from its multivariable table (OR 0.989; 95% CI, 0.979-1.000; Table S9). The age-adjusted subset (Panel B) retains a significant inverse association consistent with the main 12-study model (0.983; 0.978-0.989). The age-and-sex-adjusted subset (Panel A) is also inverse and significant but rests on four studies, two of which (Yamauchi 2025 and Du P 2026) carry 69% of the weight and reported estimates to few decimal places, and it should be regarded as exploratory. Panel B is the better-powered check.*

**Table S11. CT acquisition and Hounsfield-unit measurement methodology of the 35 included studies.**

| **Study** | **CT timing** | **CT acquisition / examination type** | **Contrast** | **Tube voltage (kVp)** | **Slice thickness** | **Level(s) measured** | **ROI site and method** | **ROI plane** | **Exclusion of cortex / venous plexus / lesions** | **Readers and reliability** |
| --- | --- | --- | --- | --- | --- | --- | --- | --- | --- | --- |
| Zhou 2021 | Preop | Preoperative lumbar/lumbosacral CT; acquisition indication NR | NR | NR | NR | L1–L4 | Vertebral body; Axial | Axial | Cortical edges, osteophytes, osseous abnormalities (including sclerosis), and disc spaces excluded | ICC 0.989 |
| Gong 2022 | Preoperative | CT including L1; acquisition region/indication NR | NR | NR | NR | L1 | L1 HU; ROI geometry and slice selection NR | NR | NR | NR |
| Lee 2022 | Preop | Preoperative CT encompassing the UIV/adjacent vertebrae; acquisition region/indication NR | NR | 120 | 3 mm | UIV (T9–12) | Vertebral body; Axial | Axial | Cortical edges and basivertebral vein avoided | NR |
| Muheremu 2022 | Preop | Preoperative lumbar/lumbosacral CT; acquisition indication NR | NR | NR | NR | Sacrum | Sacral bone; Axial | Axial | Specific exclusions NR; ROI placed within sacral cortex | NR |
| Li 2022 | Preop | Preoperative spine CT; acquisition region/indication NR | NR | 120 | NR | Fusion level | Screw holding plane; Multiplanar | Multiplanar | Cortical shell, bony endplates, and osteophytes excluded | ICC>0.86 |
| Xie 2022 | Preop | Preoperative lumbar/lumbosacral CT; acquisition indication NR | NR | NR | NR | L1–L4 | Vertebral body; Axial | Axial | Cortical margins excluded | ICC 0.925 |
| Zhang 2022 | Preoperative | Thoracic/lumbar CT encompassing the UIV; indication NR | NR | NR | NR | UIV | Vertebral body; mean of three axial ROIs | Axial | NR | Two trained orthopedic surgeons; HU-specific ICC NR |
| Hiyama 2022 | Preop | Preoperative spine CT; acquisition region/indication NR | NR | NR | NR | Cranial/caudal endplates | Endplate (cranial/caudal); Axial/Midsagittal | Axial/Midsagittal | Cortical endplate was the target; other exclusions NR | NR |
| Wu H 2023 | Preoperative | Preoperative spine CT; acquisition region/indication NR | NR | NR | NR | Superior/inferior endplates (5 regions) | Ipsilateral epiphyseal ring, central endplate; Axial | Axial | Cortical endplate was the target; other exclusions NR | Excellent (Cronbach's α >0.95) |
| Li 2023 | Preop | Preoperative lumbar/lumbosacral CT; acquisition indication NR | NR | NR | NR | L4/L5 | Screw insertion site; Axial + coronal | Multiplanar | Cortical bone and heterogeneous areas avoided | ICC 0.884 |
| Yao 2023 | Preop | Preoperative lumbar/lumbosacral CT; acquisition indication NR | Non-contrast | 120 | 3 mm | L1 | Vertebral body; Axial | Axial | Cortical margins, lateral walls, endplates, and obvious osteophytes excluded | NR |
| Zhao 2023 | Preop | Preoperative lumbar/lumbosacral CT; acquisition indication NR | NR | NR | NR | L5 | 3D trajectory; Axial | Axial | Cortical bone and basivertebral foramen avoided; hemangioma/dysosteogenesis excluded | ICC 0.88–0.92 |
| Arimura 2024 | Preop | Preoperative lumbar/lumbosacral CT; acquisition indication NR | NR | NR | NR | L4 | Vertebral body; Axial | Axial | NR | NR |
| Zhang 2024 | Preoperative (plus postoperative follow-up CT) | Lumbar CT (L4–L5); indication NR | NR | NR | NR | L4–L5 | Three oval ROIs: screw entry point, pedicle, vertebral body | Axial | Cortex excluded; venous/lesion exclusion NR | Two radiologists; HU interobserver ICC 0.627–0.875 |
| Shu 2024 | Preoperative | Preoperative lumbar/lumbosacral CT; acquisition indication NR | NR | 120 | NR | L1–L5 | Maximal-diameter ROI within the vertebral cortex; Single axial/horizontal plane at pedicle level | Axial/horizontal | NR | Two independent examiners; reliability statistic not reported; GE 32-row spiral CT at 120 kV; contrast status and slice thickness NR |
| Chen W 2025 | Preop | Preoperative lumbar/lumbosacral CT; acquisition indication NR | NR | NR | NR | L1–L5 | Vertebral body; Axial | Axial | Cortical bone, osteophytes, and non-cancellous areas avoided | ICC 0.76–0.91 |
| Chen Z 2025 | Preop | Preoperative spine CT; acquisition region/indication NR | NR | 120 | NR | Pedicle/trajectory | Pedicle cancellous; Multiplanar | Multiplanar | Cortical bone, bony endplates, posterior osteophytes, and posterior venous plexus excluded | ICC>0.84 |
| Ding 2025 | Preoperative | Lumbar CT; acquisition indication NR | NR | NR | NR | L1–L5 (L4/L5 and L1–L4 mean models) | Vertebral body; three ROIs below superior endplate, mid-body, and above inferior endplate | Axial (with median sagittal localization) | Cortex and venous plexus excluded | HU reader/reliability NR (two readers reported for VBQ only) |
| Geng 2025 | Preoperative | Lumbar CT; acquisition indication NR | NR | NR | NR | L1–L5 mean | Vertebral cancellous bone; three axial ROIs per vertebra | Axial | Cortex and vascular structures excluded | Two independent authors; HU-specific ICC NR |
| Koo 2025 | Preop | Preoperative spine CT; acquisition region/indication NR | NR | NR | NR | Adjacent vertebra | Vertebral body; Axial | Axial | Cortical margins, venous channels, and metal artifacts avoided | ICC>0.85 |
| Liu 2025 | Preop | Preoperative lumbar/lumbosacral CT; acquisition indication NR | NR | NR | NR | L1–L5 | Vertebral body; Axial | Axial | Venous plexus avoided; ROI confined to cancellous bone to minimize cortical interference | ICC>0.8 |
| Moridaira 2025 | Preop | Preoperative CT encompassing the UIV/adjacent vertebrae; acquisition region/indication NR | NR | NR | NR | UIV/UIV+1 | Vertebral body; Axial | Axial | Cortical bone and severe sclerotic abnormalities excluded | NR |
| Pennington 2025 | Preoperative | Preoperative CT encompassing the UIV/adjacent vertebrae; acquisition region/indication NR | NR | NR | NR | UIV and UIV+1 | Vertebral body; Three axial ROIs per vertebra: mid-body, immediately caudal to superior endplate, immediately cranial to inferior endplate | Axial | NR | Mean of the three HU values used; CT acquisition region, contrast status, slice thickness, HU-reader blinding, and HU interobserver reliability NR |
| Sun 2025 | Preop | Preoperative lumbar/lumbosacral CT; acquisition indication NR | NR | NR | NR | L3 | Vertebral body; Axial | Axial | Specific exclusions NR; ROI placed within cancellous bone | ICC>0.8 |
| Yamamoto 2025 | Preop | Preoperative spine CT; acquisition region/indication NR | NR | 120 | 1 mm | Trajectory | Screw trajectory; Axial | Axial | ST-HU-specific exclusions NR; ROI represented cancellous screw trajectory | ICC>0.96 |
| Yamauchi 2025 | Preop | Preoperative spine CT; acquisition region/indication NR | NR | NR | NR | Lumbar vertebra | Vertebral body (trabecular); Axial | Axial | Cortical bone, bone defects, and focal lesions avoided | NR |
| Yuan 2025 | Preop | Preoperative lumbar/lumbosacral CT; acquisition indication NR | NR | NR | NR | L1 | Vertebral body; Axial | Axial | Cortical margins excluded | NR |
| Wang Y 2025 | Preoperative | Preoperative lumbar/lumbosacral CT; acquisition indication NR | NR | NR | NR | L4 | L4 vertebral trabecular bone; Mid-sagittal | Mid-sagittal | Cortical bone and venous plexuses avoided | Not reported |
| Levy HA 2026 | Preoperative | Preoperative spine CT; acquisition region/indication NR | NR | 140 | 0.75-mm reported resolution; slice thickness NR | Operative level vertebra | Endplate (EP‑HU) + vertebral trabecular; Mid-sagittal + axial | Mid-sagittal (EP), Axial (vertebral) | venous plexus, sclerosis/lesions excluded | Good (ICC 0.86–0.84) |
| Ke 2026 | Preop | Preoperative lumbar/lumbosacral CT; acquisition indication NR | NR | 120 | 1.25 mm | L1/S1 | Vertebral body; Axial/Sagittal | Axial/Sagittal | Cortical bone, posterior venous plexus, and endplate sclerosis avoided | ICC>0.8 |
| Zhao M 2026 | Preoperative | Preoperative lumbar/lumbosacral CT; acquisition indication NR | NR | NR | NR | L1–L5 average | Vertebral cancellous bone (trabecular); Axial | Axial | Venous plexuses avoided; ROI confined to trabecular bone to minimize cortical influence | Not reported |
| Du P 2026 | Preoperative | Preoperative lumbar/lumbosacral CT; acquisition indication NR | NR | NR | NR | L1 | L1 vertebral body; L1 pedicle; Axial | Axial | Body ROI: cortex, venous plexus, bone islands, and endplate sclerosis avoided; pedicle-specific rules NR | Good (ICC >0.80) |
| Wang Y 2026 | Preoperative (postoperative CT also used for bone-loss analysis) | Standardized dedicated lumbar CT | NR | 120 | 1 mm | L1–L4 mean (L4 sensitivity measure) | Three axial vertebral-body ROIs per level; L4 mid-sagittal sensitivity ROI | Axial; mid-sagittal sensitivity measure | Cortex and venous plexus excluded | HU interobserver ICC 0.935 (L4 HU 0.971) |
| Yang 2026 | Preop | Preoperative lumbar/lumbosacral CT; acquisition indication NR | NR | 120 | NR | L3 | Screw trajectory + vertebral body; Axial | Axial | Specific exclusions NR; body and trajectory ROIs placed in cancellous bone | ICC>0.8 |
| Zhan 2026 | Preop | Preoperative lumbar/lumbosacral CT; acquisition indication NR | NR | 120 | 1 mm | L1-L4 | Vertebral body; Axial | Axial | Cortical shell, basivertebral vein, osteophytes, and sclerotic areas avoided | ICC>0.8 |

*Entries are transcribed from each study's methods section; NR, not reported. Contrast phase, tube voltage, and slice thickness were reported by a minority of studies, and the CT examination type (dedicated lumbar versus abdominopelvic or other) was frequently not stated. ROI, region of interest; UIV, upper instrumented vertebra; ICC, intraclass correlation coefficient.*

**Table S12. Derivation of the low-versus-high odds ratios displayed in Figure 3 (k = 11).**

| **Study** | **Outcome (analysis unit)** | **Threshold (source)** | **Estimate type** | **Low-HU vs high-HU events/n** | **OR (95% CI)** | **Derivation from the source article** |
| --- | --- | --- | --- | --- | --- | --- |
| Lee 2022 | UIV screw loosening (Patients (1:1 matched case-control)) | HU <126.3 (UIV) | Crude (reconstructed) | 33/57 vs 13/35 | 2.33 (0.98–5.52) | Table 3 (cut-off 126, all patients): sensitivity 71.7% (33/46), specificity 47.8% (22/46); PPV/NPV denominators give group sizes 57/35 |
| Yao 2023 | Cage subsidence or screw loosening (composite) (Patients) | L1 HU <117 | Crude (reported; cells reproduce reported CI) | 46/89 vs 24/109 | 3.79 (2.05–7.01) | Table 3 reports the dichotomised crude OR 3.789 (2.049-7.007) and adjusted OR 4.100 (2.173-7.737); crude OR is reproduced exactly by 46/43 vs 24/85 |
| Wang Y 2025 | Cage subsidence (Patients) | L4 HU <98.05 | Crude (reconstructed) | 18/31 vs 11/53 | 5.29 (2.00–14.01) | ROC cut-off 98.05: sensitivity 62.1% (=18/29), specificity 76.4% (=42/55), both integer-exact |
| Levy HA 2026 | Cage subsidence (TLIF) (Patients) | Endplate HU <369 | Adjusted (reported) | — | 5.70 (1.88–19.21) | Table 3: adjusted OR 5.70 (1.88-19.21) for total endplate HU <369 (adjusted for interbody surface area and cage material). The ROC sensitivity/specificity (printed 57.1%/79.2%; integer-exact only when read as 57/72 events and 24/42 non-events) reconstruct a crude 2x2 of 57/75 vs 15/39 (OR 5.07, 2.20-11.68). |
| Koo 2025 | Cage subsidence (OLIF) (Surgical levels (119 in 70 patients)) | HU <145.86 | Crude (reconstructed) | 22/69 vs 3/50 | 7.33 (2.06–26.17) | ROC cut-off 145.86 (level-based: 25 subsidence / 94 non-subsidence levels): sensitivity 88.0% (=22/25), specificity 50.0% (=47/94), integer-exact |
| Gong 2022 | Cage subsidence (OLIF) (Surgical segments (93 in 73 patients)) | L1 HU <110 | Crude (reported) | — | 9.50 (1.90–46.20) | Table 3: univariable OR 9.5 (1.9-46.2) and multivariable OR 9.0 (1.3-64.2) for L1 HU <110; no group counts reported |
| Ke 2026 | Pedicle screw loosening (PLIF) (Patients) | S1 HU <225.63 | Crude (reconstructed) | 50/122 vs 11/163 | 9.60 (4.72–19.53) | Table 7 ROC cut-off 225.63: printed sensitivity 67.86%/specificity 81.97% resolve to integers only when swapped (81.97% = 50/61 events; 67.86% = 152/224 non-events) |
| Yuan 2025 | Distal pedicle screw loosening (Patients) | L1 HU <169 | Adjusted (reported) | — | 10.67 (2.06–55.19) | Table 4: adjusted OR 10.672 (2.064-55.188) for HU <169 (backward-elimination multivariable model); no group counts or sensitivity/specificity reported |
| Moridaira 2025 | Proximal junctional failure (Patients) | UIV HU <98 | Crude (direct counts) | 11/15 vs 6/31 | 11.46 (2.69–48.87) | Text reports PJF 11/15 (HU <98), 4/15 (98-134), 2/16 (>=134) directly |
| Ding 2025 | Cage subsidence (Patients) | Mean L1-L4 HU <125.72 | Crude (reconstructed) | 87/121 vs 15/105 | 15.35 (7.82–30.16) | ROC cut-off 125.72: sensitivity 85.3% (=87/102), specificity 72.6% (=90/124), integer-exact |
| Chen Z 2025 | Pedicle screw loosening (Patients (left L5 screw)) | Left pedicle HU <71.25 | Crude (reconstructed) | 16/28 vs 1/45 | 58.67 (7.05–488.15) | Table 6 (left pedicle cut-off 71.25): printed sensitivity 0.786/specificity 0.941 resolve to integers only when swapped (0.941 = 16/17 events; 0.786 = 44/56 non-events) |

Every odds ratio shown in Figure 3 was derived from the source article as follows. Where a study reported an odds ratio for the dichotomised HU variable, that estimate was used (Gong 2022 and Yao 2023, crude; Yuan 2025 and Levy HA 2026, adjusted). Otherwise a crude 2×2 table was reconstructed from reported counts (Moridaira 2025) or from reported sensitivity and specificity, but only where both proportions resolve exactly to integer event and non-event counts at the study's stated denominators; in several articles the printed sensitivity and specificity correspond to the complication-free or high-HU class as the positive class, which is noted. Confidence intervals for reconstructed tables use the standard large-sample formula for the log odds ratio. Studies whose threshold-level data could not be reconstructed exactly (Muheremu 2022, Du P 2026, Yamauchi 2025, Wu H 2023), that reported no threshold (Geng 2025), no confidence interval (Yamamoto 2025), no HU-group counts (Zhang 2024), or a three-level classification (Chen W 2025) are carried as directional evidence in Table S5. Analysis units other than patients (surgical levels or segments) are stated; within-patient clustering was not accounted for in the source articles.

**Supplementary Figures**

**Figure S1.** Subgroup forest plots of the main continuous adjusted-effect model, stratified by mechanical-complication category. Squares are study-specific adjusted odds ratios per 1-Hounsfield-unit increase, sized by random-effects weight; horizontal lines are 95% confidence intervals; diamonds are subgroup summary estimates (REML τ² with Hartung–Knapp–Sidik–Jonkman intervals). Screw loosening: k = 3, OR 0.980 (95% CI, 0.949–1.012). Cage subsidence: k = 5, OR 0.982 (95% CI, 0.971–0.993). Proximal junctional kyphosis/failure: two studies shown at study level without a summary diamond (not pooled). Corresponding numeric estimates are given in Table S8.

**Figure S2. Funnel plot with Duval–Tweedie trim-and-fill for the 12-study continuous adjusted-effect model. Filled circles are observed studies and open circles the studies imputed by trim-and-fill (L0 estimator; side of imputation selected from the data); the vertical line is the trim-and-fill-adjusted estimate and dashed lines the 95% pseudo-confidence limits. Five studies were imputed to the right of the observed estimate, giving an adjusted OR of 0.986 (95% CI, 0.982–0.989) compared with the observed 0.983 (0.978–0.989); the direction and significance of the association were unchanged. The asymmetry is more plausibly attributable to between-study heterogeneity than to publication bias, although small-study effects cannot be excluded.**
